# Supplementary material for: β-Adrenergic signaling induces Notch-mediated salivary gland progenitor cell control
Source: Stem Cell Reports. 2021 Oct 21;16(11):2813–24. doi: 10.1016/j.stemcr.2021.09.015 (PMC8581054; doi:10.1016/j.stemcr.2021.09.015)
Supplement: Document S1. Figures S1–S3 and Tables S1–S4, and supplementary methods [file mmc1.pdf]

Stem Cell Reports, Volume *16*

Supplemental Information

β-Adrenergic signaling induces Notch-mediated salivary gland progenitor cell control

X. Wang, P. Serrano Martinez, J.H. Terpstra, A. Shaalan, G.B. Proctor, F.K.L. Spijkervet, A. Vissink, H. Bootsma, F.G.M. Kroese, R.P. Coppes, and S. Pringle

Notch-mediated salivary gland progenitor cell inhibition by β-blockers

Supplementary Figures

Fig. S1

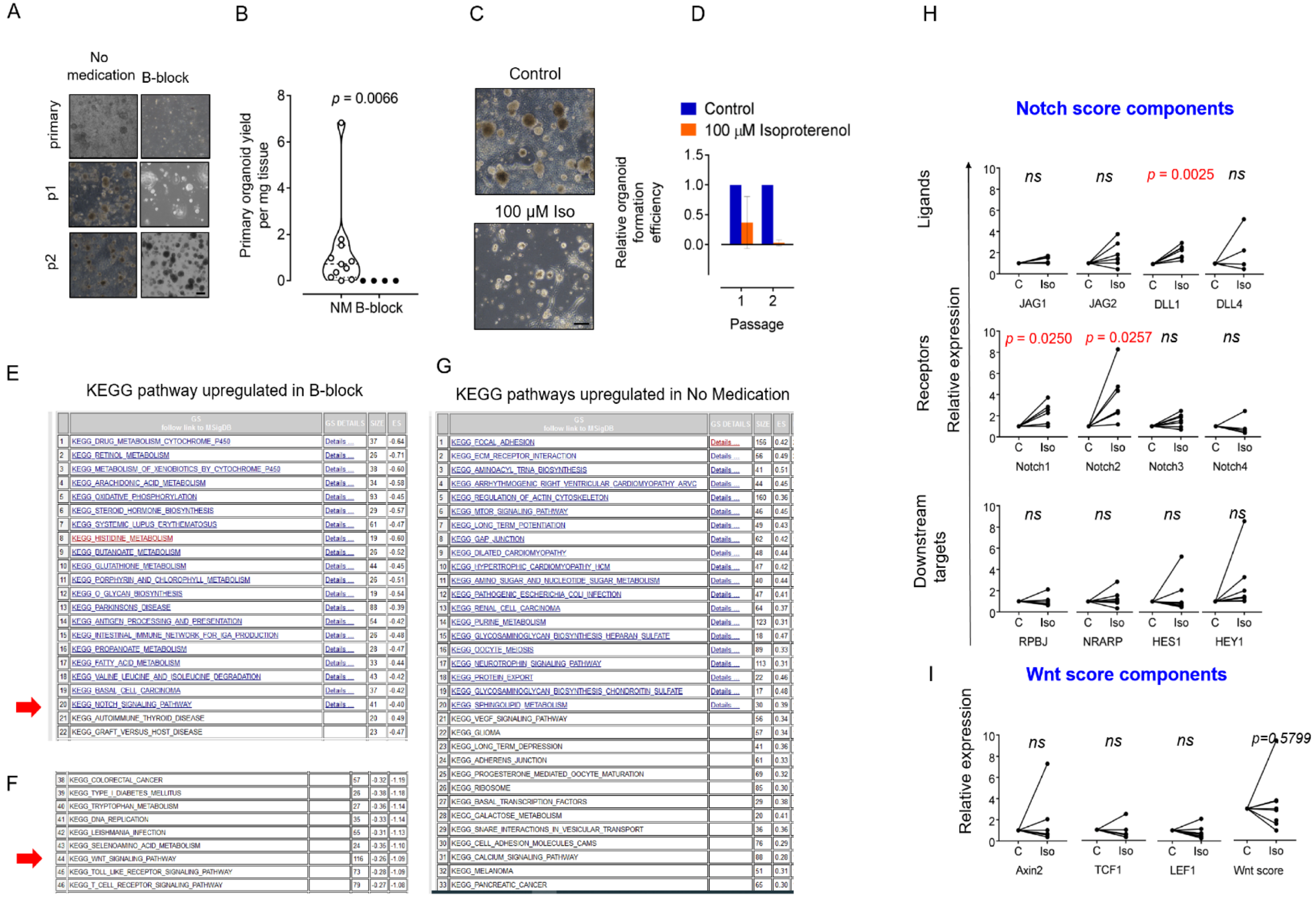

Fig. S2.

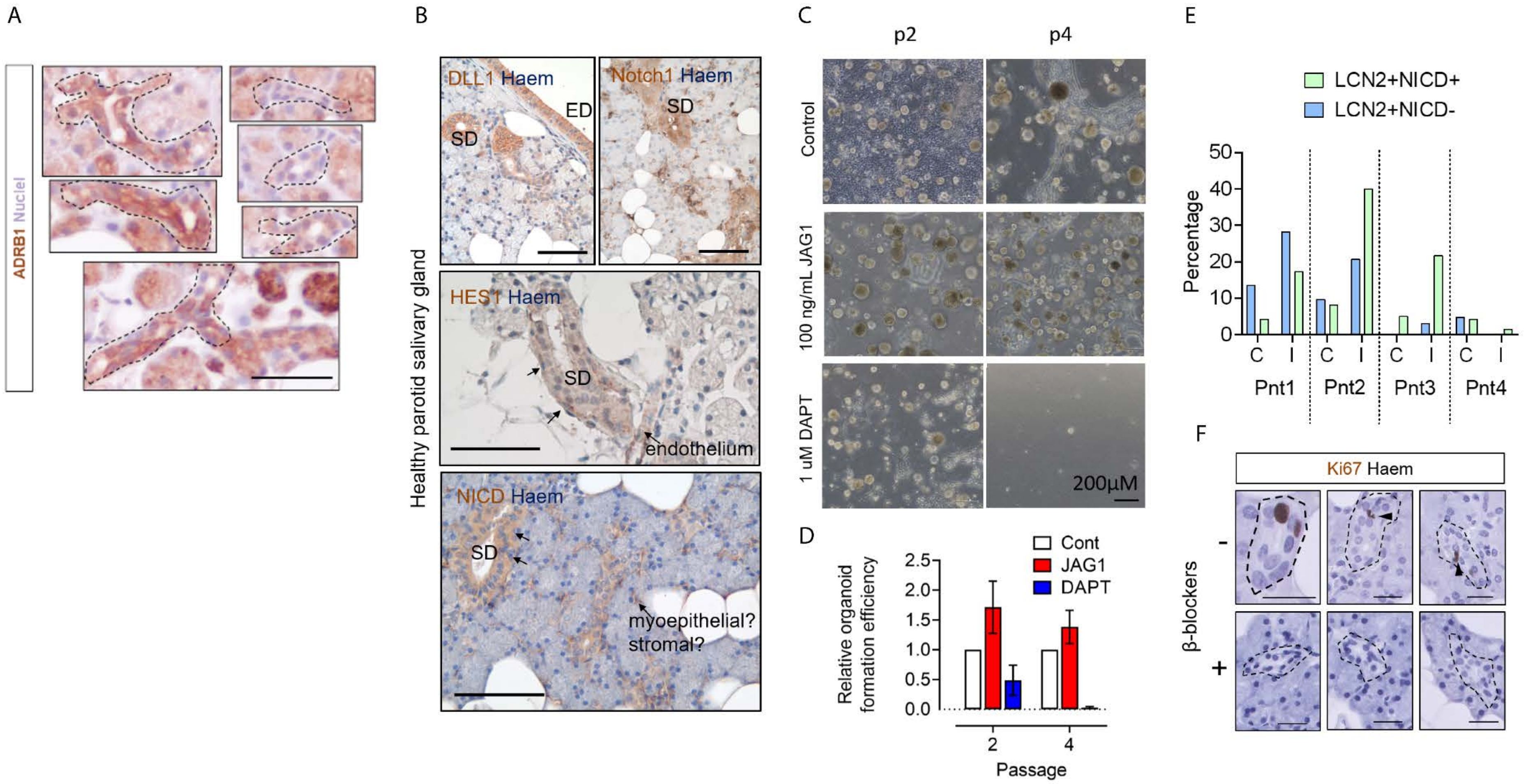

A
ADRB1 Nuclei
B
DLL1 Haem
ED
SD
Notch1 Haem
SD
HES1 Haem
SD
endothelium
NICD Haem
SD
myoepithelial?
stromal?
Healthy parotid salivary gland
C
p2
p4
Control
100 ng/mL JAG1
1 uM DAPT
200µM
D
Relative organoid formation efficiency
Cont
JAG1
DAPT
2.5
2.0
1.5
1.0
0.5
0.0
2
4
Passage
E
LCN2+NICD+
LCN2+NICD-
Percentage
50
40
30
20
10
0
C
I
Pnt1
Pnt2
Pnt3
Pnt4
F
Ki67 Haem
β-blockers
-
+

Fig. S3

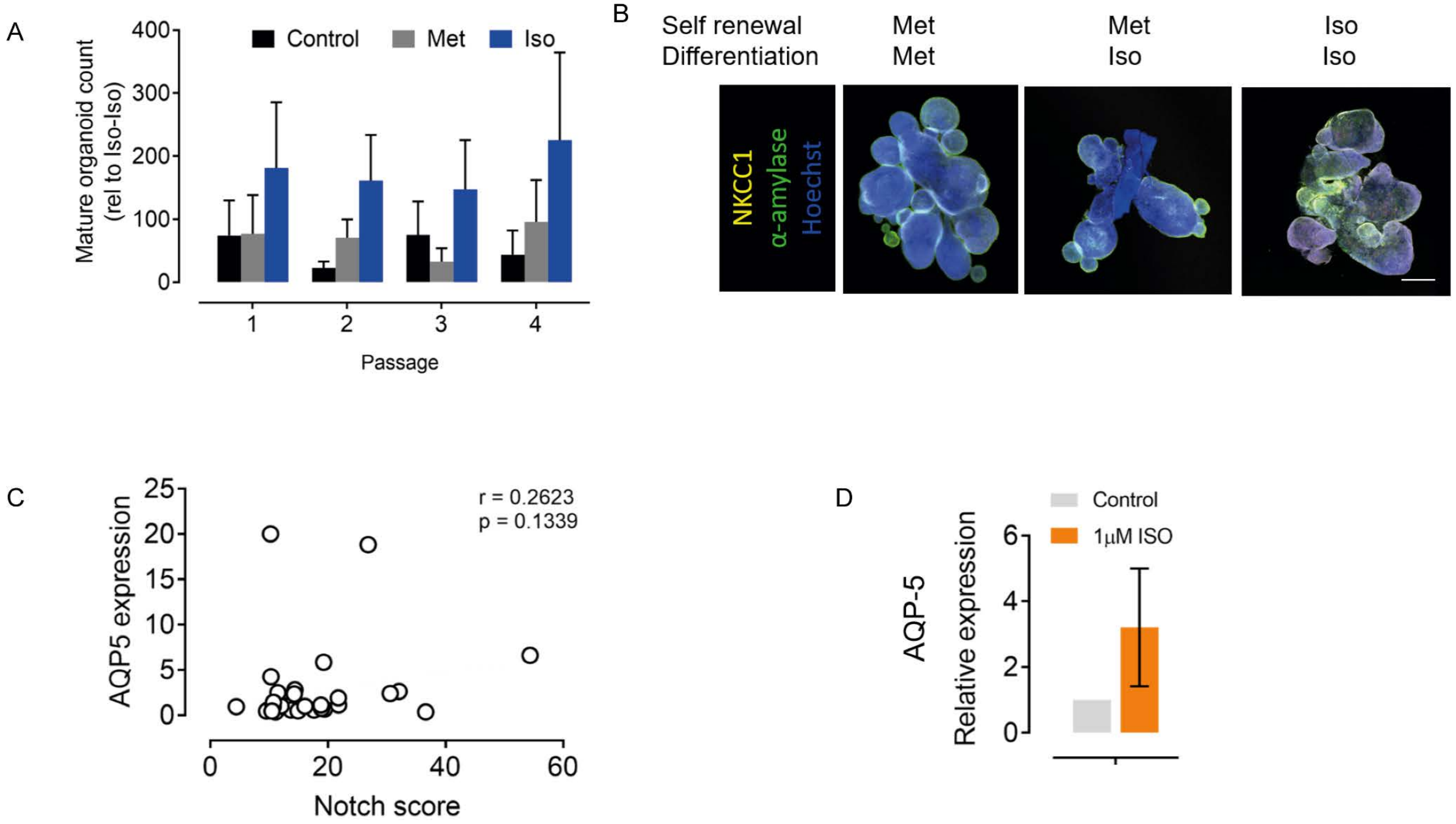
A
Mature organoid count
(rel to Iso-Iso)
400
300
200
100
0
Control
Met
Iso
1
2
3
4
Passage
B
Self renewal
Differentiation
Met
Met
Met
Iso
Iso
Iso
NKCC1
α-amylase
Hoechst
C
AQP5 expression
25
20
15
10
5
0
r = 0.2623
p = 0.1339
0
20
40
60
Notch score
D
Control
1μM ISO
AQP-5
Relative expression
6
4
2
0

Supplementary Figure legends

Fig. S1. *Salivary gland organoid yield from biopsies of a patient taking β-blocking drugs was initially low. RNASeq analysis shows upregulation of Notch signaling when cultures following β -blocker use recover in organoid formation efficiency*. *The Notch pathway in salivary gland organoids is activated by isoproterenol, whereas the Wnt pathway is not.* A) Phase contrast microscopy of organoid cultures generated from biopsies harvested from patients taking β-blocking drugs (B-block) in primary cultures, passage 1 and passage 2, compared to biopsies from patient not taking any medication (No medication). B) Quantification of organoid forming efficiency in primary culture. Each data point represents a separate patient. NM = No medication. C) Phase contrast microscopy of SGPC cultures in control and 100 µM isoproterenol conditions. D) Quantification of 100 µM Isoproterenol supplementation. *n* = 3 separate patient isolations. Bar represents mean, error bars represent standard deviation. E) Gene Set Enrichment Analysis (GSEA) using KEGG pathway gene sets, and identifying pathways upregulated in p2 organoids from patient taking a *β*-blocking drug. Red arrow denotes ranking of Notch signaling pathway (left panel). F) Position of Wnt pathway in upregulated genes p2 organoids from patient taking a *β*-blocking drug. G) GSEA identifying pathways upregulated in healthy control organoids. Tables in E show number of genes in gene set (size), expression score (ES), normalized ES (NES). *p* values are not shown, due to statistical invalidity of comparing one biopsy from a patient taking β-blocking drugs to 6 controls. H) Relative expression of Notch score components, grouped by ligands, receptors and downstream targets. I) Relative expression of Wnt score components and total Wnt score. In all figures, paired *t*-testing was performed. Each paired data set represents a separate patient isolation. Raw *p*-values are given when significant. ns = no significance.

Fig. S2. *ADRB1 expression decreases in some intercalated ducts following β-blocker use*. *DLL1, Notch1, HES1 and NICD are expressed by parotid SG striated duct cells. Incubation of human parotid SGOs with the Notch ligand JAG1 induces a subtle increase in the number of SGOs, whereas inhibiting the Notch pathway reduces all organoid formation* A) Expression of ADRB1 in intercalated ducts (outlined with dashed lines) in a patient following β-blocking drug use. All images are from the same tissue sample. Scale bar = 50 µM. B) Healthy parotid salivary gland immunostained for DLL1,

Notch1 and HES1, showing expression of DLL1 and Notch1 in both basal and luminal striated duct layers. DLL1 was additionally expressed in excretory ducts, Notch1 in scattered stromal cells, and HES1 in basal striated duct cells (arrows) and endothelial cells. C) Phase contrast microscopy of SGOS cultures at passages 2 and 4, in control, 100 ng/mL JAG1 and 1 µM DAPT conditions. D) Quantification of control, JAG1 and DAPT incubations. $n = \geqslant 5$ separate patient isolations at each passage and for each group. E) Percentage of SGO cells expressing LCN2 and NICD when cultured in control conditions, or with isoproterenol. Pnt = Patient. F) Ki67 staining of tissue from a patient taking β-blockers (+) and in control SG tissue (-).

Fig. S3 *Supplementary human parotid mature SGO formation data* A) Quantification of mSGO formation efficiency without isoproterenol exposure in self-renewal assay conditions. *n* = 4, 9, 5 and 4 for passages 1,2,3 and 4, respectively. Each data point is a separate biological replicate B) Whole mount staining of mSGOs for expression of NKCC1 and α-amylase in culture conditions containing metoprolol or isoproterenol, as indicated. C) Correlation between AQP5 expression and Notch scores in mSGO differentiation assays. Data points represent the expression of AQP5 from 2 patients from passages 1 to 4 in differentiation. D) Expression of AQP5 in self-renewal assays exposed to 1µM isoproterenol. *n*=7, each data point is a separate biological replicate.

Supplementary Tables

Supplementary Table 1

List of medications taken by non ß-blocker-exposed ‘healthy SG’ donors, and additional medication taken by patients taking β-blockers.

Healthy salivary gland donor	Medications used
1	Atorvastatin, carbasalate calcium, clopidogrel, ezetimib, pantoprazol, paracetamol, perindopril**
2	Aspirin, Furosemide*, pantoprazol, amlodipine, candesartan, diazepam*, diclofenac
3	Spiriva, vitamine B12 injections
4	Acetylcysteine, omeprazol, brimonidine*
5	Simvastatin, chloramphenicol, dexamethasone eye drops, diclofenac eye drops,
6	Hydrochlorothiazide
7	Diclofenac, levothyroxine, omeprazol
8	None
9	None
10	None
11	Dextran hypromellose eyecream, triptoreline, paracetamol
12	Propranolol*
Patients taking β-blockers	Additional medications used
1	Seretide, Salbutamol, Euthyrax, Omeprazole, vit-D, Ascal, Atorvastatin, paracetamol codeine, Fexofenadine, eye cream, Dermovate vaginal, artificial tears.
2	talbutamine, metformine, hydrochlorothiazide, lisinopril*, prednisolone, acenocoumarol, simvastatine, slow K, Omeprazol, psyllium vezels, brinzolamide, artelac, vidisec carbogel, Budesonide (anti-inflammatory).
3	Omeprazole
4	Plaquenil, Bromazepam, Vidisec, Carbogel, Aprovel.
5	Triamtereen, Tiotropium, Omeprazole, Naproxen, Irbesartan, Insuline glargine, Dextra 70/hypromellose oogdruppels, Ciclesonide aerosol
6	Ezetimibe

Table S1. Details of medication used by representative patients donating healthy salivary gland biopsies, and those taking β-blockers. . Medications indicated with asterixes have potential for interaction with salivary gland function according to Wolff et al, Drugs R D., 2017 17(1): 1-28. * High / moderate levels of evidence, ** weak evidence.

Supplementary Table 2

Clinical characteristics of patients taking ß-adrenoreceptor blocking medication analyzed in this study.

Patient	β-blocking medication	Age	Sex	Resting secretion speed ml/min			Stimulated secretion speed ml/min			Analysis performed	Used in
				PR	PL	Sub+SubL	PR	PL	Sub+SubL		
1	Metoprolol Citalopram*	62	F	0	0.004	0.08	0.08	0.08	0.21	SGO culture	FigS1, Fig1
2	Metoprolol Amitriptyline*	74	F	0	0	0.02	0.03	0.02	0.02	SGO culture	FigS1, Fig1
3	Metoprolol Citalopram*	58	F	0	0	0.04	0	0	0.07	SGO culture	FigS1, Fig1
4	Duloxcetine Escitalopram	48	F	0.01	0.01	0.24	0.01	0.26	0.39	SGO culture & RNASeq	FigS1, Fig1
5	Metoprolol	66	F	0.1	0.09	0.44	0.18	0.17	0.42	Immunohistochemistry	Fig1B-D
6	Metoprolol	62	F	0	0	0.01	0.01	0.02	0.05	Immunohistochemistry	Fig1B-D

Table S2. Clinical characteristics of patients taking ß-blocking medication, from which biopsies were studied. Abbreviations: PR = parotid right; PL – parotid left; Sub+SubL = submandibular and sublingual salivary gland secretion combined; SGO = salivary gland organoid culture. Stimulated secretion speeds were measured using citric acid. Additional drugs capable of causing hyposalivation according to Wolff et al, (Drugs R D., 2017 17(1): 1-28) are marked with an asterix. * High / moderate levels of evidence

Supplementary Table 3

Gene name	Gene Symbol	Forward primer	Reverse primer	Amp size
glyceraldehyde-3-phosphate dehydrogenase	GAPDH	tcttttgcgtcgccagccgag	cccgttctcagccttgacggtg	234
Jagged1	JAG1	aggccgttgctgacttagaa	gcagaagtgggagctcaaag	230
Jagged2	JAG2	gtcaaggtggagacggttgt	tggtagagcacgtccttgtg	250
Delta-like ligand 1	DLL1	tgtgcctcaagcactaccag	ttctgttgcgaggtcatcag	230
Delta-like ligand 4	DLL4	acctttgggtgtctgtctgg	acttttggaaacacggatgc	209
Notch Receptor 1	NOTCH1	actgtgaggacctggtggac	ttgtaggtgttggggaggtc	196
Notch Receptor 2	NOTCH2	atgactgccctaaccacagg	ccagccgttgacacatacac	206
Notch Receptor 3	NOTCH3	atcttggggcctaaagaga	gactgagagggtgggtggta	202
Notch Receptor 4	NOTCH4	ctaggggctcttctcgtcct	caacttctgcctttggcttc	178
NOTCH regulated ankyrin repeat protein	NRARP	gactcaattcgaacccgaaa	acttccatgaaggggaaacc	220
Recombination signal binding protein for immunoglobulin kappa J region	RBPJ	cgcattattggatgcagatg	caggaagcgccatcatttat	171
hes family bHLH transcription factor 1	HES1	ctctcttccctccggactct	aggcgcaatccaatatgaac	186
hes related family bHLH transcription factor with YRPW motif 1	HEY1	cgaggtggagaaggagagtg	ctgggtaccagccttctcag	177
Aquaporin-5	AQP5	actgggttttctgggtaggg	gtggtcagctccatggtctt	184
α-amylase	AMY	tgtcagggctgagtgttctg	ttccggtataaatgcgaacc	198
Na-K-2Cl cotransporter 1	NKCC1	tcagtcagccatacccaaagg	cagtggcatctcgaacaacac	107
axis inhibition protein 2	Axin2	cctgccaccaagacctacat	cttcattcaaggtggggaga	241
transcription factor 1	TCF1	gcagggtctagaaggctgtg	acctgctctaccagccagaa	242
lymphoid enhancer binding factor 1	LEF1	aacatggtggaaaacgaagc	gggttggcagtgattgtctt	201

Supplementary Table 4

Target	Target species	Abbrev.	Host	Supplier	Catalog #	Clone	Dilution
**Primary antibodies**							
β-1 adrenergic receptor	human	ADRB1	Rabbit	ThermoFisher Scientific	PA1-049	Polyclonal	1:50
DLL1	human	-	Rabbit	Novus Biologicals	NBP2-27088SS	Polyclonal	1:100
notch 1	human	-	Mouse	Santa Cruz Biotechnology	11-651-C025	mN1A	1:1000
HES1	human	-	Rabbit	Abcam	ab71559	Polyclonal	1:50
Notch intracellular domain	Human	NICD	Rabbit	Abcam	Ab8387	Polyclonal	1:1000
cytokeratin 7	human	K7	Mouse	Merck	MAB3226	RCK105	1:100
Ki67	human	-	Rat	ThermoFisher Scientific	14-5698-82	SolA15	1:100
NKCC1	human	-	Goat	Abcam	ab99558	Polyclonal	1:75
α-amylase	human	-	Sheep	ThermoFisher Scientific	PA1-85176	Polyclonal	1:50
cytokeratin 14	human	K14	Mouse	Merck	CBL197	LL002	1:100
Lipocalin-2	Human	LCN2	Rabbit	Merck	HPA002695	Polyclonal	1:50
smooth muscle actin	human	SMA	Mouse	DAKO	M085129-2	1A4	1:100
aquaporin 5	human	AQP5	Rabbit	Abcam	ab92320	EPR3747	1:200
**Secondary antibodies**							
Rabbit and mouse IgG UltraVision HRP polymer			N/A	DAKO	TL-125-HL	N/A	Ready-to-use
Mouse and rabbit IgG, AP and HRP conjugated, double staining kit			N/A	ThermoFisher Scientific	TL-012-MARH	N/A	Ready-to-use
Mouse IgG (H+L) Alexa Fluor 488 conjugated			Goat	ThermoFisher Scientific	A-11001	N/A	1:1500
Rabbit IgG (H+L) Alexa Fluor Plus 594 conjugated			Donkey	ThermoFisher Scientific	A32754	N/A	1:1500
Goat IgG (H+L) Alexa Fluor Plus 647 conjugated			Donkey	ThermoFisher Scientific	A32849	N/A	1:1500
Rat IgG (H+L) Alexa Fluor 647 conjugated			Goat	ThermoFisher Scientific	A-21247	N/A	1:1500
Sheep IgG–FITC conjugated			Donkey	Merck	F7634-1ML	N/A	1:40
Mouse IgG, biotin conjugated			Rabbit	DAKO	E0433	N/A	1:300
Rabbit IgG, biotin conjugated			Goat	DAKO	E0432	N/A	1:300

Supplementary Methods

Quantification and statistical analysis

Methods used for quantification are described in relevant sections. Data was tested for normality using Shapiro-Wilk test. Two-Way ANOVAs were used to test for significant differences, if n numbers were matched across groups and data was parametric. Dunnett's post-hoc testing was used for multiple comparisons, to establish between which groups significant differences lay. If uneven *n* numbers were present across groups, a mixed effects model was used, with subsequent Tukey's post-hoc testing for multiple comparisons. If data was non-parametric, a Kruskal-Wallis test with Dunn's post-hoc testing was used. Adjusted *p*-values for each analysis are given in the relevant text section. Statistical analysis was performed using GraphPad Prism 8.02.

qPCR analysis

Total RNA as was extracted from cultured cells as appropriate using the RNeasy Microkit (Qiagen), including DNase incubation, as per manufacturer's instructions. One µg of total RNA was reverse transcribed to cDNA using 0.5 µg oligo$(dT)_{15-18}$ primers, 1.0 mM dNTPs, 1X Reaction Buffer, 20U Ribolock and 200 U of RevertAid Reverse Transcriptase (all Thermo Fischer Scientific), in a total volume of 20 µL per reaction. cDNA product was diluted ten-fold in water and used at this concentration for qPCR. qPCR was performed using SsoAdvanced Universal SYBR Green qPCR Mastermix (Biorad), with primers at a final concentration of 500 nM from a 10 µM stock. 2.5 µL of diluted cDNA was used per reaction, and all reactions were performed in triplicate, in a total volume of 10 µL. Primer sequences can be found in Supplementary Table 3. A 2-step qPCR cycle with the BioRad iCycler qPCR machine was used for target amplification according to SSoAdvanced Universal SYBR Green Mastermix instructions, and CFX Manager software for analysis. Expression of all 12 members of the Notch pathway, relative to GAPDH, was summed to derive the 'Notch score'. The Wnt score was derived from addition of relative expression of 3 Wnt pathway genes.

RNASeq analysis of SGOs

Total RNA was extracted from salivary gland organoids from a patient taking β-blockers and 6 controls by using Absolutely RNA Miniprep kit (Agilent Technologies, CA. Cat: 400800) followed the manufacturer's recommendations. The integrity of RNA was

examined by Agilent 2100 bioanalyzer. Subsequent sequencing was performed by using SMART-Seq v4 Ultra Low Input RNA Kit (Clontech, Cat: 634890) and Nextera XT DNA Library Prep Kit (Illumine, Cat: FC-131-1096) followed manufacturer's recommendations. Prepared DNA libraries were sequenced on HiSeq 2500 System. Gene set enrichment analysis (GSEA) was performed using GSEA software version 4.0.2, using a defined KEGG gene set for pathway analysis (tp.broadinstitute.org://pub/gsea/gene_sets/c2.cp.kegg.v6.2.symbols.gmt). Owing to *n*=1 nature of biopsy analyzed, no statically valid calculation of p values could be performed. Data can be accessed via the NCBI Sequence Reads Archive (accession number PRJNA506620).

Paraffin section staining

For immunostaining of human tissue, formalin-fixed paraffin embedded 4µm sections of human parotid salivary gland underwent antigen retrieval with EDTA buffer (pH = 8) for 15 min. A double staining kit from Thermofisher was used, according to manufacturer's instructions. Primary antibodies and concentrations are detailed in the Key Resources table. Sections were imaged using the Olympus BX50 microscope. After dehydration, the tissue was paraffin-embedded and sectioned at 4um thickness. The tissue sections were dewaxed, boiled for 8 min in pre-heated 10mM citric acid (Sigma-Aldrich) retrieval buffer pH 6.0, containing 0.05% Tween20. Primary and secondary antibodies were as in Supplementary Tables 4 Labelling was amplified using an avidin-biotin-horse radish peroxidase complex (VECTASTAIN® Elite® ABC HRP Kit Peroxidase, Standard, PK-6100) and the diaminobenzidine (DAB) chromogen (SIGMAFAST™ 3,3 DAB Tablets). Nuclear staining was performed with hematoxylin. Images were acquired with Leica DM6 B microscope using LAS X software.

Whole mount staining mSGOs

Samples were released from culture gels using 1mg/mL Dispase as per passaging protocol and fixed in cold 4 % PFA for 40 minutes. Samples were then washed in PBS and blocked in 1 % BSA 0.5 % Triton for 15 min at room temperature (RT). Samples were allowed to settle, and blocking buffer removed carefully. Primary antibodies were then added at concentrations stated in Supplementary Table 4, in 1 % BSA 0.5 % Triton, and incubated overnight at 4 ºC . To wash SGOs/mSGOs, staining buffer was removed as much as possible and replaced with fresh staining buffer, and left to

incubate for 1 hour at RT. This was repeated 3 times. Secondary antibodies were added at final concentrations of 1:1500 in 0.2 % BSA, 0.5 % Triton, and again incubated overnight at 4 ºC (supplementary Table 6). Washing steps were repeated as above. After the final wash, as much washing buffer was removed as possible, and structures incubated for 20 minutes at RT in clearing solution, before removal from staining well and mounting according to Dekker et al protocol (40). Structures were imaged on the Leica TCS SP8 microscope, including composition of maximum projection and tiled images.

Frozen sections SGOs and staining

SGOs harvested from self-renewal plates in the control and isoproterenol treated groups were fixed in cold 4 % PFA for 40 minutes, following by washing with 1%BSA/PBS twice. After that, SGOs were incubated in 20% sucrose for 2 hours, until SGOs sunk to the bottom of the eppendorf. The majority of the sucrose was removed, leaving approximately 50µL left. The SGOs were then placed in a mould filled with approximately 150µL Tissue-Tek® O.C.T.™ Compound and incubated at -20 ºC for at least 20 min. 5 µm thickness of sections SGOs were prepared. SGOs sections were firstly fixed with 4% PFA for 5 min, then incubated with primary antibodies (ADRB1, cytokeratin 7, HES1 and Ki67, as shown in the Supplementary Table 4). Slides were carefully washed and secondary antibodies added as shown in the Supplementary Table 6, and counterstained with Hoechst for visualizing the nuclei. Structures were imaged on the Leica TCS SP8 microscope, including composition of maximum projection and tiled images.

Cell smear staining protocol

Samples to be analyzed were processed to single cells using Dispase and Trypsin as described in the self-renewal protocol. Cells were fixed in 4 % PFA for 20 minutes at RT, before washing with PBS, and resuspending in 15uL PBS. Gelatin-coated microscopy slides were prepared by submerging slides in a 0.3 % (w/v) solution of type A gelatin (275 Bloom; Sigma) containing 0.05% (w/v) chromium potassium sulphate (Sigma), at 40-50 ºC degrees Celsius. Slides were allowed to dry, and cell suspension smeared over the surface of the slide using a P100 pipette tip. When dried, smeared cells were circled with a wax pen, and cells incubated with primary anti LCN2, or NICD antibodies (Supplementary Table 4) overnight at 4 ºC. Following washes in PBS, cells

were incubated in appropriate secondary antibodies and subsequent flourophores provided in the Tyramide Signal Amplification Superboost kit (Thermofisher) washed again in PBS, and mounted in Faramount mounting medium. Cell smears were imaged using the Leica TCs SP8 confocal microscope. At least 4 fields of randomly selected microphotographs under 630x magnification in each group were quantified. The proportion of $LCN2^+$, $NICD^+$ and $LCN2^+NICD^+$ cells were calculated by the amount of $LCN2^+$, $NICD^+$ and $LCN2^+NICD^+$ cells divided by total cells (determined by the number of nuclei).
