# Supplementary material for: β-Adrenergic signaling induces Notch-mediated salivary gland progenitor cell control
Source: Stem Cell Reports. 2021 Oct 21;16(11):2813–24. doi: 10.1016/j.stemcr.2021.09.015 (PMC8581054; doi:10.1016/j.stemcr.2021.09.015)
Supplement: Document S2. Article plus supplemental information [file mmc2.pdf]

Stem Cell Reports
Article

OPEN ACCESS

# β-Adrenergic signaling induces Notch-mediated salivary gland progenitor cell control

X. Wang,[1] P. Serrano Martinez,[2,3] J.H. Terpstra,[1] A. Shaalan,[4] G.B. Proctor,[4] F.K.L. Spijkervet,[5] A. Vissink,[5] H. Bootsma,[1] F.G.M. Kroese,[1] R.P. Coppes,[2,3] and S. Pringle[1,*]
[1]Department of Rheumatology and Clinical Immunology, University of Groningen, University Medical Center Groningen, Groningen, the Netherlands
[2]Department of Biomedical Sciences of Cells and Systems, University of Groningen, University Medical Center Groningen, Groningen, the Netherlands
[3]Department of Radiation Oncology, University of Groningen, University Medical Center Groningen, Groningen, the Netherlands
[4]Centre for Host and Microbiome Interactions, King's College London, London, UK
[5]Department of Oral and Maxillofacial Surgery, University of Groningen, University Medical Center Groningen, Groningen, the Netherlands
*Correspondence: s.a.pringle@umcg.nl
https://doi.org/10.1016/j.stemcr.2021.09.015

## SUMMARY

β-Adrenergic signaling blockade is a mainstay of hypertension management. One percent of patients taking β-blockers develop reduced salivary gland (SG) function. Here we investigate the role of SG progenitor cells in β-blocker-induced hyposalivation, using human SG organoid cultures (SGOs). Compared with control SGs, initial low SG progenitor cell yield from patients taking β-blockers was observed. When passaged, these SGOs recovered self-renewal and upregulated Notch pathway expression. Notch signaling was downregulated *in situ* in β-adrenergic receptor-expressing luminal intercalated duct (ID) cells of patients taking β-blockers. Control SGOs treated with β-adrenergic agonist isoproterenol demonstrated increased proportion of luminal ID SGO cells with active Notch signaling. Control SGOs exposed to isoproterenol differentiated into more mature SGOs (mSGOs) expressing markers of acinar cells. We propose that β-blocker-induced Notch signaling reduction in luminal ID cells hampers their ability to proliferate and differentiate into acinar cells, inducing a persistent hyposalivation in some patients taking β-blocking medication.

## INTRODUCTION

Inhibiting β-adrenergic signaling using β-blockers is commonly employed to treat hypertension, resulting in heartbeat slowing and blood vessel widening. Dependence on β-blockers for hypertension management increased between 2000 and 2010 by 5%, with the β-blocker metoprolol being the sixth most prescribed drug in the United States in 2018 (Bloch, 2016; Kane, 2019). Although effective in hypertension management, β-blocker use is accompanied by a plethora of side effects; for example, headaches, diarrhea, and fatigue. Data suggest that 1% of patients taking metropolol will suffer from xerostomia as a result of salivary gland (SG) function loss, with metropolol monotherapy leading to a 2.86-fold increased risk of dry mouth development (Djukić et al., 2015; Glick et al., 2020; Wolff et al., 2017). Although perhaps sounding inconsequential, persistent dry mouth leads to difficulties with speaking, eating, and swallowing; persistent dental issues; and dramatically reduced patient quality of life. This hyposalivation would seem to result initially from a direct β-blocker-induced reduction in SG acinar cell function. β-blocker administration reduces sympathetic-nerve-mediated glandular stimulation, causing reduced secretion of total salivary protein and α-amylase, and can reduce unstimulated whole/parotid gland specific saliva flow (Cowman et al., 1994; de Matos et al., 2010; Speirs et al., 1974; van Stegeren et al., 2006). Similar results have been found in animal models studying the acute effects of β-blockers or loss of sympathetic autonomic signaling through β-adrenergic blockade (Johnson and Cortez, 1988; Matsuo et al., 2000). The resultant diminished volume and protein content culminates in less lubrication, and accompanying difficulties such as speaking and swallowing (Nederfors, 1996; Speirs et al., 1974; van Stegeren et al., 2006). Any effect of β-blockers on other SG cell types is not known.

Stimulation of β-adrenergic receptors by isoproterenol increases cyclic adenosine monophosphate (cAMP), and subsequent activation of cAMP-dependent protein kinase A signaling triggers amylase secretion (Purushotham et al., 1992; Selye et al., 1961). In rats, the parotid SG swells to five times its size within 2–3 weeks of isoproterenol administration with concurrent increased proliferation of acinar cells (Humphreys-Beher et al., 1987). Second, a less well-explored effect is that of adrenergic signaling on SG ductal cells. Isoproterenol administration, interestingly, also induced ductal cell proliferation in rat SGs (Hand and Ho, 1985). Expression of diverse cell growth, proliferation, and survival genes in rat SG has also been reported post isoproterenol treatment (Yeh et al., 2012; Zhou et al., 2015), although whether these pertain to the acinar or ductal cell compartment is unclear. Chronic loss of sympathetic autonomic innervation leads to significant atrophy of parotid glands (Proctor et al., 1988), but the effect of chronic application and withdrawal of β-blockade on function of the SG is not substantiated. These data leave a substantial gap in our understanding of the permanence of β-blocker-induced hyposalivation.


This is an open access article under the CC BY license (http://creativecommons.org/licenses/by/4.0/).

The homeostasis of the SG is likely maintained by heterogeneous populations of SG progenitor cells (SGPCs). Current literature would suggest their residence in the basal layers of the striated ducts (SDs), in the intercalated ducts (IDs), and within the acinar cell population themselves (Aure et al., 2015; Maimets et al., 2016; Xiao et al., 2014). Considering the effect of β-adrenergic signaling on ID cells in the rat, a role for β-adrenergic signaling in human SGPC dynamics is feasible. Although activity of glial-derived neurotrophic factor (GDNF), Wnt, Notch, and Yap/TAZ pathways is crucial for SGPC function, the intricacies of SGPC control have not yet been fully resolved (Dang et al., 2009; Hwang et al., 2014; Maimets et al., 2016; Peng et al., 2017; Xiao et al., 2014). The importance of neuronal stimuli for maintenance of SGPC populations in the mouse has previously been clearly demonstrated, and the parasympathetic nervous system (PNS) specifically shown to maintain a population of epithelial progenitor cells in the embryonic mouse SG (Knox et al., 2010).

Probing progenitor cell involvement in disease contexts can be achieved using organoids (Clevers, 2016). Organoids are formed by proliferation of tissue-specific progenitor cells, for example SGPCs, which differentiate into cell lineages comprising the organ in question (Clevers, 2016). Generation of SG organoids (SGOs) from human SG biopsies has been employed to demonstrate the likely replicative senescence of SGPCs in primary Sjögren syndrome (pSS), an autoimmune disease also partly characterized by SG function loss (Pringle et al., 2019; Wang et al., 2020a; Wang, et al., 2020b).

In this study, we aimed to investigate the effect of β-adrenergic signaling on SGPC dynamics, with a view to understanding the development of SG dysfunction following β-blocker use, and also improving our comprehension of SGPC biology.

## RESULTS

### β-Adrenergic signaling release of blockade increases SGO formation efficiency and upregulates Notch pathway activity

To perform preliminary assessment of the effect of adrenergic signaling blockade on SGPCs, we cultured primary SGOs from parotid gland biopsies of patients taking β-blockers (n = 4) (clinical characteristics in Tables S1 and S2). No primary SGOs reaching our minimum size criteria of 50 μm diameter could be isolated from these biopsies, although single and small clusters of cells were present (Figures S1A and S1B). This is in contrast to SGOs from parotid gland tissue of control individuals, from which the primary organoid efficiency was 1.3 ± 0.58 SEM organoids/mg biopsy tissue (Figures S1A and S1B). SGPC ability to proliferate after this stage can be assessed by calculating organoid formation efficiency. Primary cultures from patients taking β-blockers and control cultures were further maintained in culture conditions promoting SGPC proliferation (self-renewal assay) until passage 2, which we considered a presumptive β-blocker wash-out period (~3 weeks). By the end of this period, SGPCs from patients taking β-blocking drugs recovered organoid-forming potential (13% ± 2% standard deviation), surpassing that of organoids from control glands (4% ± 4% standard deviation; Figure 1A).

The transcriptome of these bulk passage 2 SGOs from a patient taking a β-blocking drug (13% organoid formation efficiency *in vitro* at passage 1, 10% at passage 2); see clinical characteristics in Tables S1 and S2) was compared with that of six control biopsies unexposed to β-blockers (mean organoid-forming efficiency at passage 2 of 3%). A total of 13,154 differentially expressed genes was detected. These genes belonged to 160 Kyoto Encyclopedia of Genes and Genomes (KEGG) pathways. Eighty-two of these 160 pathways were upregulated in organoids from subjects on β-blockers compared with mean expression in controls. The first 19 upregulated pathways comprised a majority of metabolic processing pathways (top three were retinol metabolism, cytochrome p450 metabolism, and metabolism of xenobiotics by cytochrome p450). The 22$^{nd}$ upregulated pathway, and the first pathway with connections to stem and progenitor cells, was the Notch pathway (Figure S1C). Of the 47 genes comprising the KEGG Notch signaling pathway gene set list, 24 (51%) were upregulated in organoids derived from the patient taking a β-blocker, compared with mean of organoids from six control biopsies (Figure 1B). These 24 genes included three Notch ligands (DLL1, JAG1, JAG2), three Notch receptors (Notch1, Notch3, Notch4) and two Notch downstream targets (HES1, HEY1), and 15 other regulatory elements of the Notch pathway (Figure 1B). A known important progenitor cell pathway, the Wnt pathway, was also enriched in SGO cultures after β-blocker wash-out, at upregulated KEGG pathway position 44 (Figure S1D). Compared with passage 2 organoids from the patient using β-blocker, no stem/progenitor cell pathways were upregulated in organoids without β-blocker use (Figure S1E). From these preliminary data and previous animal studies, we suggest that SGPCs may be at least partially controlled by β-adrenergic signaling, via the Notch pathway.

### β-Adrenergic agonist isoproterenol increases Notch signaling in SGOs

SGO cultures derived from control SGs and exposed *in vitro* to the β-adrenergic agonist isoproterenol (Iso) demonstrated a significant increase in relative organoid formation efficacy compared with untreated cultures (Figures 1C and 1D). A maximum of 2.5-fold ±0.5 SEM increase was

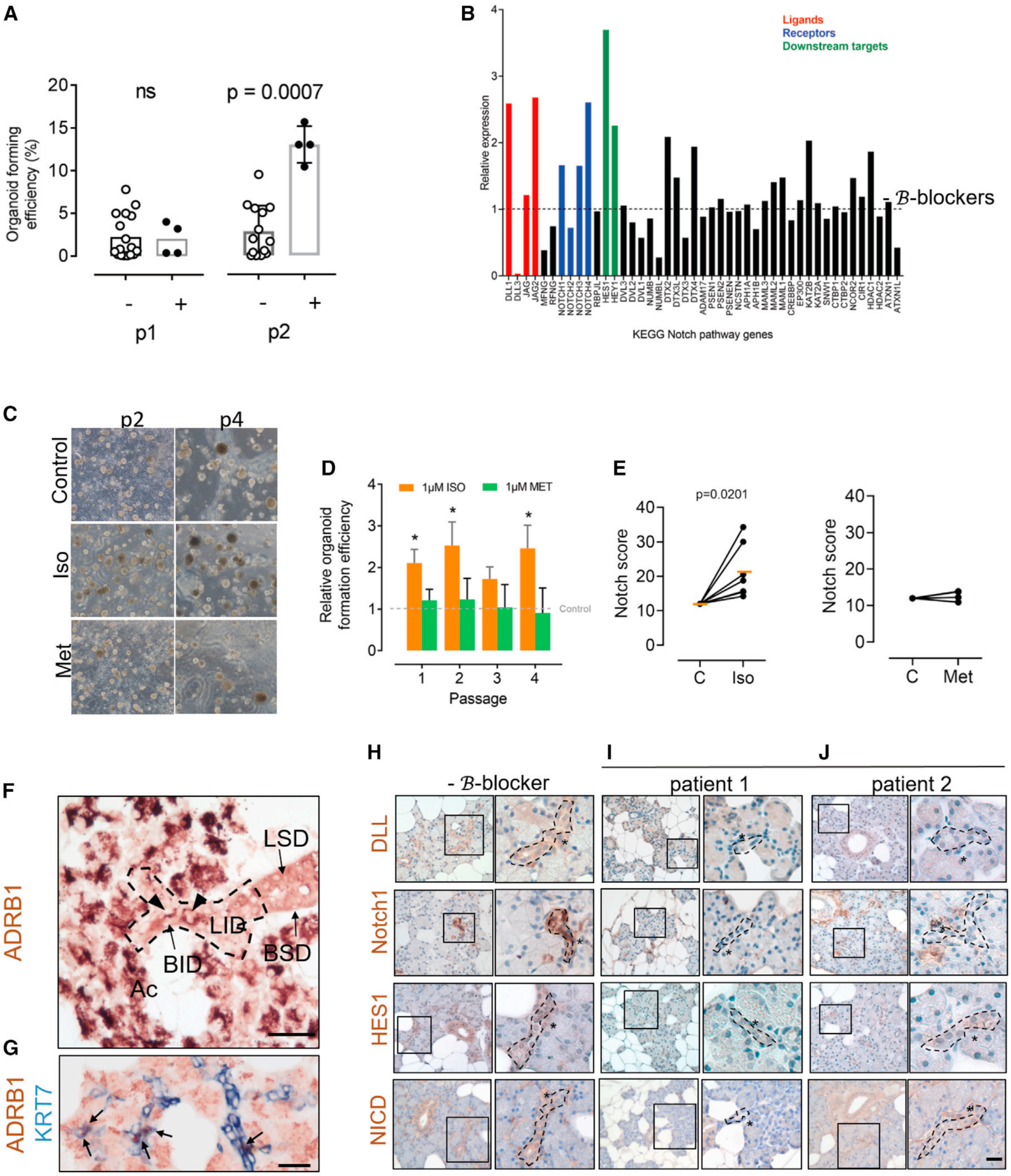


**Figure 1. Effects of β-adrenergic signaling on SGO proliferation and Notch signaling. Luminal ID cells express ADRB1 and members of the Notch signaling pathway**

(A) Quantification of organoid-forming efficiency in passages 1 and 2. Each data point represents a separate patient. Bars represent mean. Kruskal-Wallis non-parametric statistical testing was used; raw p value is given.

(B) Relative expression of 47 members of KEGG Notch signaling pathway gene set in passage 2 SGOs from a patient taking β-blockers, compared with mean of expression in six no-medication controls. Notch ligand, receptors, and downstream targets are highlighted in colors stated.

(C) Representative phase contrast microscopy of SGPC culture as SGOs, at passages 2 and 4. Control SGOs were maintained under control conditions or with Iso (Iso) or Met, stimulation conditions. Scale bar represents 200 μm.

*(legend continued on next page)*

observed following 1 μM Iso exposure, compared with untreated control cultures without Iso (Figures 1C and 1D; adjusted p values 0.02, 0.047, and 0.047 at passages 1, 2, and 4 respectively). Higher Iso dosing (100 μM), similar to that used in the literature, appeared to be toxic to SGO cultures (Figures S1F and S1G) (Srinivasan et al., 2016). Interestingly, when SGOs from control SGs were incubated with 1 μM β-blocker metropolol (Met), no significant effect on organoid formation efficiency was observed (Figures 1C and 1D), potentially due to the lack of stimulation of adrenergic receptors in absence of Iso.

In order to examine the direct effect of β-adrenergic signaling on the Notch pathway, we performed qPCR for expression of members of the Notch pathway, on SGOs from healthy SGs, following Iso exposure. These Notch pathway members comprised four Notch ligands (JAG1, JAG2, DLL1, DLL4), four Notch receptors (Notch1–4), and four Notch downstream targets (RBPJ, NRARP, HES1, HEY1; Figure S1H) Expression of each gene in Iso-exposed SGO cultures was normalized to its matched untreated control SGO culture (given a value of 1). A Notch score of these relative expression values was generated by summing all 12 relative gene expressions. Iso exposure resulted in a 1.8-fold significant upregulation in Notch pathway activity when comparing control (by definition a Notch score of 12) with Iso-treated SGOs at p2 (Notch score of 21 ± 8 standard deviation; Figure 1E). The Notch score was not significantly changed following Met addition to control SGOs, in line with an inactive β-adrenergic signaling system (Figure 1E). In our RNA sequencing (RNA-seq) data, members of the Wnt pathway, which also plays a role in stem/progenitor cell dynamics, were present among the top 50 upregulated genes in organoids, after a β-blocker wash-out period in culture. Addition of Iso to the SGO cultures, however, did not affect activity of the Wnt pathway in our SGOs system, whereby a Wnt score (sum of relative expression of three Wnt pathway genes, Axin2, TCF1, and LEF1) of 3.5 ± 3 standard deviation was found in Iso-treated SGOs at p2, compared with a Wnt score of 3 in controls (Figure S1I).

## β-blocker use downregulates Notch signaling in luminal intercalated duct cells of the parotid SG

To determine which cells in the human parotid SG may be capable of processing β-adrenergic signals, we performed immunostaining for the β1 adrenoreceptor (ADRB1) in tissue sections from control parotid glands. ADRB1 is a receptor for the neurotransmitter norepinephrine and also the receptor to which commonly prescribed β-blockers, such as Met, bind. ADRB1 immunopositivity was observed, as expected, in acinar cells, considering the role of β-adrenergic stimulation in saliva secretion (Figure 1F). Luminal intercalated duct (ID) cells cells also expressed ADRB1, in contrast to lack of expression in basal ID cells, basal SD, and luminal SD cells (Figure 1F). Keratin 7 (K7) can be used to label luminal ID cells (basal ID cells are KRT7 negative) (Pringle et al., 2020b). Colocalization of ADRB1 and K7 expression in luminal ID cells confirmed expression of ADRB1 by luminal ID cells (Figure 1G). In tissue from a patient taking β-blocker medication, ADRB1 expression was maintained in some luminal ID cells, and appeared reduced in others (Figure S2A). Our RNA-seq and qPCR results suggest that β-adrenergic signaling may influence the activity of the Notch pathway. In order to map expression profiles of members of the Notch signaling pathway, we performed immunostaining for the Notch pathway members DLL1 (ligand for Notch1), Notch1 (receptor for DLL1, DLL3, JAG1, and JAG2), and HES1, and Notch intracellular domain (NICD; both Notch downstream targets) in control SG tissue of patients that did not use β-blocker. Luminal and basal ID and SD cells all robustly expressed DLL1, Notch1, HES1, and NICD (Figures 1H and S2B). Acinar cells rarely expressed these Notch signaling markers. Non-epithelial cell populations, including stromal and endothelial cells, also expressed members of the Notch signaling pathways (Figure S2B). Interestingly, only weak expression of DLL1, Notch1, HES1, and NICD was observed in luminal and basal ID cells from age-matched patients taking β-blockers (Figures 1I and 1J; clinical characteristics in Table S1). Expression of Notch pathway members did not

(D) Quantification of relative SGO formation efficiency in presence of β-adrenergic stimulation (Iso) or blockade (Met). Compared with control set at 1 (gray dashed line). Minimum biological replicates (SGOs derived from separate patients) at each passage for each group are as follows: Iso = 10, Met = 5. Statistical analysis was performed with mixed effects ANOVA. Asterisk indicates analysis relative to control group. $^*p < 0.05$.

(E) qPCR data showing the Notch score in control passage 2 SGO cultures, and those incubated with Iso or Met. Statistical analysis is paired t test. Each pair of data points represents a separate patient isolation.

(F) Control human parotid gland tissue immunostained for the ADRB1 adrenergic receptor. Ac, acinar cells; LID, luminal ID cell; BID, basal ID cell; BSD, basal SD cell; LSD, luminal SD cell. Arrowheads denote $ADRB1^+$ LID cells. Scale bars represent 50 μm.

(G) Double immunohistochemical staining of ADRB1 and Keratin 7 (K7). Scale bars represent 50 μm. Arrows denote ADRB1/K7 double-positive cells.

(H) DLL1, Notch1, HES1, and NICD expression in control parotid SG tissue (minus β-blocker).

(I and J) DLL1, Notch1, HES1, and NICD expression in two patients taking the β-blocker drug Met (plus β-blocker). In (H)–(J), the second image for each patient and protein examined represents high-resolution magnification of boxed inset. Dashed line denotes luminal ID cells. Asterisk highlights basal ID cells.

seem to be as affected in luminal or basal SD cells, where DLL1 and NICD expression was still present following β-blocker use. β-Blocker use thus seems to coincide with reduced Notch signaling in both luminal and basal ID cells of the human parotid SG.

### β-Adrenergic signaling promotes proliferation of luminal ID-like cells via activation of the Notch signaling pathway

SGOs represent a heterogeneous culture of SG ductal progenitors. According to our immunohistochemical data, the only ductal cells expressing β-adrenergic receptors are luminal ID cells. In order to unravel the importance of ADRB1 signaling for luminal ID cells, we probed our SGO system. Although K7 and KRT14 can distinguish luminal ID cells from basal ID cell in tissue sections, they cannot distinguish these cells from their counterparts in the SDs, where basal SD cells also express K14, and luminal SD cells K7 (Figure 2A). Luminal ID cells in parotid SG tissue, however, express the growth factor and iron chelator lipocalin 2 (LCN2), whereas basal ID and both luminal and basal SD cells do not (Figure 2B, summarized in Figure 2C). We also detected the presence of $K7^{+}ADRB1^{+}$ cells in control SGO cultures and those treated with Iso, suggesting the presence of luminal ID cells in our SGO cultures (in relation to histology in Figures 1G) and a potential of SGOs to respond to adrenergic stimulation (Figure 2C). To probe the response of the Notch pathway in luminal ID cells to Iso stimulation in SGOs, we used LCN2 as a marker of luminal ID cells, and the downstream Notch target NICD as a readout of Notch pathway activity. Iso treatment induced a 3.6-fold increase in the proportion of $LCN2^{+}NICD^{+}$ cells in SGOs to 20% ± 8% SEM, compared with untreated controls (6% ± 1% SEM; Figures 2E and 2F). The proportion of $LCN2^{+}NICD^{+}$ cells following Iso treatment was approximately 50% less than following treatment with the Notch ligand JAG1, used as a positive control (42% ± 12% SEM; Figures 2E, 2F, and S2C–S2E). The proportion of $LCN2^{+}NICD^{-}$ cells in control SGOs was 7% ± 3% SEM, while in the Iso and JAG1 treatment groups it was 13% ± 7% SEM and 26% ± 8% SEM, respectively, suggesting also that luminal ID cells may proliferate directly in response to adrenergic signaling, in addition to initiating Notch signaling in neighboring cells (Figure S2E). Inhibition of the Notch pathway in SGOs by incubation with the γ-secretase inhibitor (N-[N-(3,5-difluorophenacetyl)-l-alanyl]-S-phenylglycine t-butyl ester) (DAPT) decreased organoid forming to 0.49 ± 0.26 SEM of untreated cultures at passage 2 (Figures S2C and S2D). These data suggest that Notch pathway activation may be transiently activated by Iso and JAG1, and potentially exerts an effect on luminal ID cell proliferation.

The Notch signaling pathway functions through cell-cell contact. In serial SGO section immunostaining (5 μm thickness), we detected $K7^{+}ADRB1^{+}$ potential Notch signal-giving cells (cell 1; Figure 2G) adjacent to putative $HES1^{+}Ki67^{+}$ Notch signal-receiving cells (cell 2; Figure 2G). Based on co-expression of K7 and ADRB1 in tissue (Figure 1G), this signal-giving cell is likely to be a luminal ID cell, signaling to a neighboring SGO cell to induce Notch signaling. Interestingly, cell 2 was $K7^{-}ADRB1^{-}$, suggesting that other SGO cell types apart from luminal IDs are capable of interacting with luminal ID cells.

These data combined suggest that $LCN2^{+}$ luminal ID-like cells in parotid SGOs undergo Iso stimulation and upregulate Notch signaling in neighboring cells.

### β-Adrenergic signaling primes SGO cells for differentiation into mature SGOs containing secretory units

In order to investigate the effect of β-adrenergic signaling on SGO cell differentiation, we employed our mature SGO (mSGO) formation assay. SGOs from control individuals were first generated in control or Iso-exposed self-renewal conditions, where, as we have shown above, the Notch pathway is upregulated in luminal ID-like cells. SGOs were then transferred into differentiation conditions. In control conditions, large, multi-branched structures formed spontaneously after 14 days in differentiation (Figure 3A). True differentiation of SGOs into a secretory unit containing mSGOs was shown first by qPCR for NKCC1 and α-amylase (acinar cell genes). Expression of NKCC1 and α-amylase mRNA increased in mSGOs compared with SGOs ($p = 0.0043$ for NKCC1 expression; Figure 3B). No expression of NKCC1 or α-amylase protein was detected in SGOs from self-renewal culture when analyzed using whole-mount staining (Figure 3C). In contrast, both NKCC1 and α-amylase were detected in mSGOs (Figure 3D). mSGOs were quantified, with minimum criteria for an mSGO being at least three branches with end buds. Figure 3E shows an overview of wells in representative differentiation assay in different medium combinations, where large structures are visible. Following Iso pre-treatment in self-renewal assays, significantly more mSGOs were formed when Iso was also added to differentiation medium (Iso-Iso), compared with control medium (passage 2, $p = 0.0012$; passage 3, $p = 0.0015$; Figure 3F). Adding Met to differentiation cultures previously exposed to Iso in self-renewal conditions generated significantly less mSGOs than Iso-Iso conditions (passage 2, $p = 0.0008$; Figure 3F). mSGOs were formed in Iso-Iso conditions with a mean efficiency of 7% ± 13% standard deviation of organoids seeded, with a tendency to demonstrate greater mSGO formation in earlier passages (Figure 3G). For comparison, mSGOs formed spontaneously in conditions with control

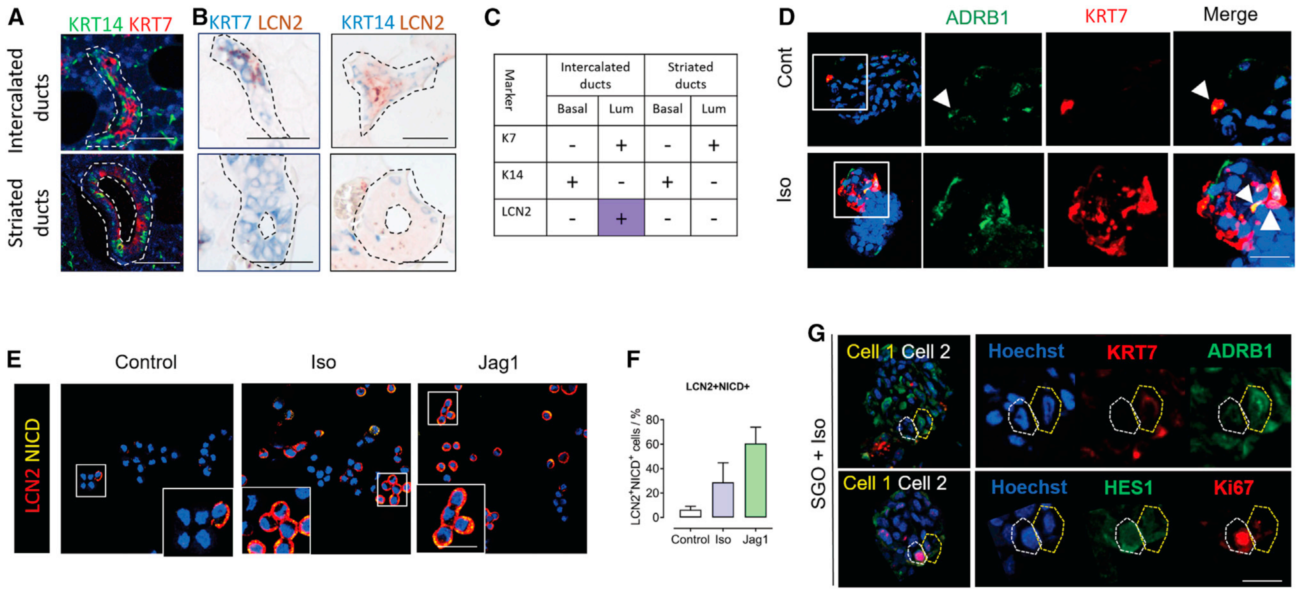


**Figure 2. β-Adrenergic signaling induces proliferation of luminal ID-like cells in SGOs via activation of the Notch pathway**

(A) Double immunofluorescence staining of healthy parotid SGs for Keratin 7 (K7) and Keratin 14 (K14). White dotted line demarcates the intercalated or SD outlines as appropriate. Scale bars represent 50 μm.

(B) Double immunohistochemical staining of healthy parotid gland for K7 and the luminal ID cell marker LCN2. Black dashed lines outline intercalated or SDs as appropriate. Scale bars represent 50 μm.

(C) Table summarizing K7, K14, and LCN2 expression profiles of cell types in the adult human parotid gland.

(D) Immunostaining for ADRB1 and K7 in SGOs from healthy SGO cultures using control medium or with Iso supplementation. Scale bar represent 25 μm.

(E) LCN2 and the Notch pathway downstream target NICD immunofluorescence staining of SGO cells in control, Iso, or JAG1 stimulated conditions. Insets show LCN2$^+$NICD$^+$ cells from area in white box, as examples of cells quantified. Scale bar for inset boxes represents 25 μm.

(F) Quantification of proportion of LCN2$^+$NICD$^+$ cells following Iso or JAG1 exposure, compared with matched controls. n = 4 separate patient isolations.

(G) Serial sections of Iso treated SGOs, immunostained for K7, ADRB1, the Notch pathway downstream target HES1, and Ki67. Putative Notch signal sending cells (cell 1) is outlined in yellow, and Notch signal receiving cells (cell 2) in white. Nuclei are counterstained with Hoechst in (D)–(F). Scale bar represents 25 μm.

medium in both self-renewal and differentiation steps at a frequency of 0.7% ± 1.7% standard deviation. In conditions where no Iso was administered in self-renewal conditions, a trend for increase mSGO formation efficiency with Iso in differentiation conditions was observed, but this was not significant (Figure S3A).

We noticed that some mSGO cultures generated from exposure to Iso in both self-renewal and differentiation conditions expressed higher levels of acinar cell marker proteins (Figure S3B). The Notch score calculated via qPCR from differentiation cultures correlated with summed expression of the acinar cell genes NKCC1 and α-amylase from the same cultures ($r = 0.72$; $p < 0.0001$), suggesting, together with the immunostaining, that an active Notch signaling pathway is associated with differentiation of SGPCs in mSGO assays (Figure 3J). Interestingly, no correlation was found between the Notch score of differentiation cultures and AQP5 expression, another gene expressed by acinar cells ($r = 0.26$; $p = 0.1339$; Figure S3C). This finding implies that AQP5 may mark a pro-acinar cell population or a subset of cells not responsive to Notch signaling, or a population of cells downstream of β-adrenergic signaling but β-adrenergic signaling and not activated by the Notch pathway in the same manner. Pro-acinar cells have indeed been suggested to exist by other groups (Aure et al., 2015; Weng et al., 2018). Expression of AQP5 increased in self-renewal cultures following Iso administration, suggesting a progenitor or pro-acinar step-like function of AQP5$^+$ cells in an organoid culture system (Figure S3D). Active Notch signaling in mSGOs was further confirmed by whole-mount staining, where expression of the Notch downstream target HES1 colocalized with the acinar markers α-amylase and NKCC1 and structures (Figure 3I). ADRB1 expression in mSGOs also colocalized with α-amylase and NKCC1, mirroring ADRB1

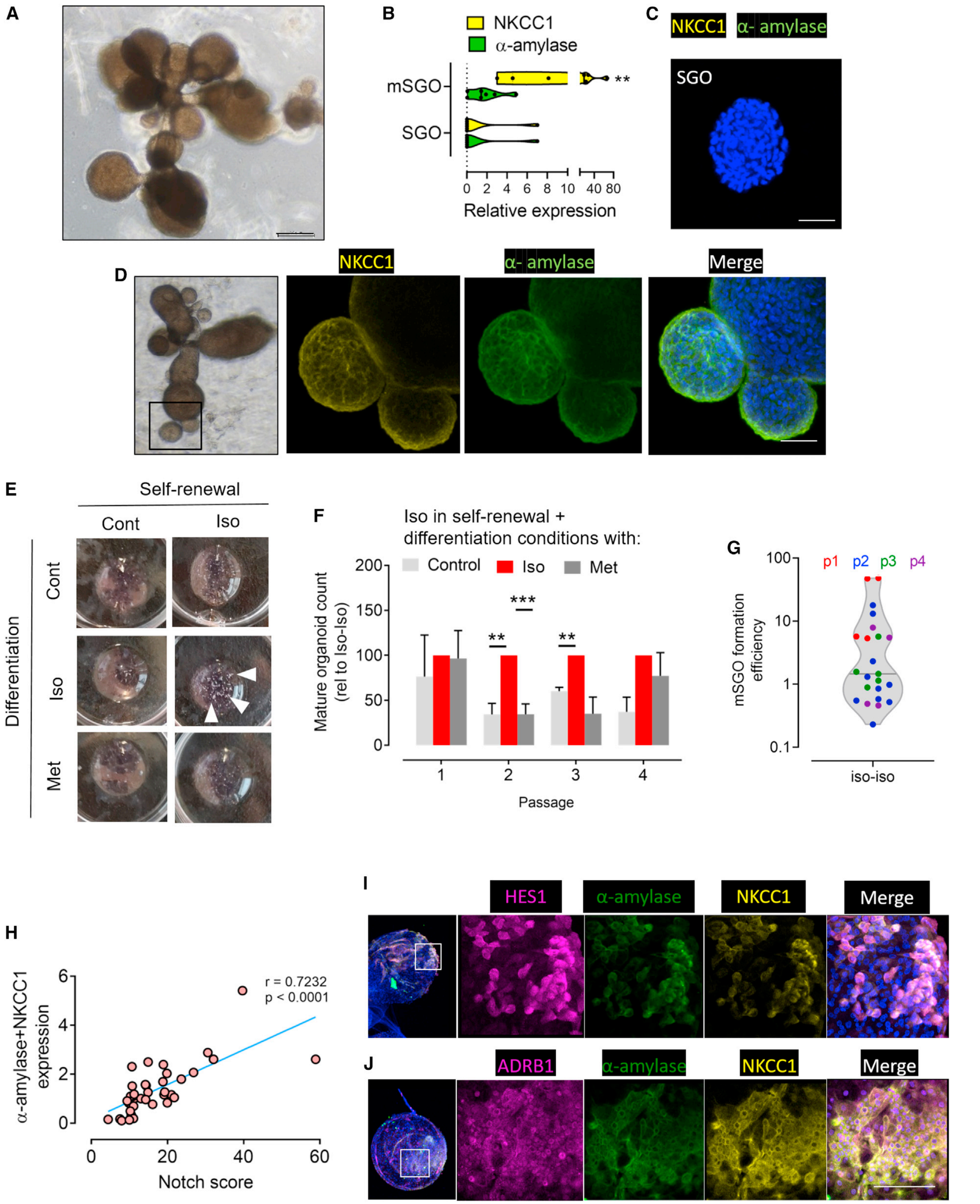


*(legend on next page)*

expression in acinar cells in parotid tissue (Figure 3J). These data suggest that β-adrenergic signaling leads to differentiation of SGOs into mSGOs, requiring activation of the Notch pathway.

## DISCUSSION

Reduced salivary volume, protein content, and α-amylase activity following blockade of β-adrenergic signaling is well established (Cowman et al., 1994; de Matos et al., 2010; Speirs et al., 1974; van Stegeren et al., 2006) and has been attributed to inhibition of saliva production by acinar cell. Earlier studies demonstrated that both the PNS and sympathetic nervous system (SNS) play roles in the long-term maintenance of SGs, since chronic loss of each nerve supply leads to glandular atrophy (Proctor and Carpenter, 2007). The cholinergic-signal-based PNS, which provides the main drive for salivary secretion, has been demonstrated convincingly by several groups to occupy a central role in SG progenitor cell dynamics. In the murine embryonic setting, stimulation of epithelial Keratin $5^+$ progenitor cells by the PNS maintains the epithelial stem/progenitor cell population and embryonic SG development (Knox et al., 2010). Treatment using neurotrophic factor neurturin can protect and restore the PNS function and increase murine SG epithelial regeneration (Knox et al., 2013). Studies detailing the involvement of the SNS branch are limited to one. In human parotid SG cells in culture, expression of acinar cell differentiation markers increased following exposure to Iso (sympathetic signaling agonist) and carbachol (parasympathetic agonist) (Srinivasan et al., 2016). These initial cultures were characterized as expressing Keratins 5 and 14, implying a possible ductal cell type, although which exactly is not clear. Cells were also stimulated simultaneously with Iso and carbachol, prohibiting the isolation of the effect purely of Iso (Srinivasan et al., 2016). Our data build on this work and demonstrate the ability of β-adrenergic receptor signaling, a constituent of the SNS, to induce proliferation of a potential population of luminal ID progenitor cells and to enhance differentiation of SGOs into mSGOs.

The complete system supporting SG homeostasis has yet to be fully resolved, with progenitor cells residing potentially in the basal SD, ID, and acinar cell niches (Aure et al., 2015; Kwak et al., 2018; Maimets et al., 2016; van Luijk et al., 2015; Weng et al., 2018). Both the SDs and IDs are comprised of two layers of cells: a basal and a luminal layer. This study is the first to separate the two often-combined layers of the ID and define a role for the luminal ID layer in human parotid gland homeostasis. We observed a subtle increase in SGO formation efficiency with Iso stimulation, in line with the minimal numbers of luminal ID-like cells present. As Notch pathway components are also expressed by basal SD cells, which are likely to comprise a large proportion of SGO cells, we expected to find a more sizable increase of proliferation when SGOs were stimulated with the Notch ligand JAG1. This was not the case. This observation may imply that Notch1 expression does not necessarily reflect functionality, and that more robust stimulation is needed for the full activation of Notch pathway, or indeed combined stimulation with the β-adrenergic pathway. Broad inhibition of Notch signaling with DAPT all but obliterated our SGO cultures, implying indeed that the Notch pathway must be both active and salient to SGPC functionality. Although not capable of inducing large-scale SGO proliferation, JAG1 incubation did appear to induce a switch in identity of the cells toward Notch-activated luminal ID-like cells ($LCN2^+NICD^+$), from 6% to 42% of total cells.

Some debate exists as to the identity of cells contained in SGO cultures, which we acknowledge is also applicable to

**Figure 3. β-Adrenergic-mediated Notch signaling primes SGOs for differentiation into mSGOs containing acinar cells**
(A) Phase contrast microscopy of representative mSGO. Scale bar represents 200 μm.
(B) Acinar marker genes NKCC1 and α-amylase expression in SGOs and mSGOs. Each data point represents a separate patient culture.
(C) Whole-mount immunostaining of SGO for NKCC1 and α-amylase. Scale bar represents 50 μm.
(D) NKCC1 and α-amylase whole-mount immunostaining in mSGO, including low-resolution view of the stained mSGO. Scale bar represents 50 μm.
(E) Photographs of differentiation assays in conditions with and without Iso exposure in self-renewal (top row), followed by control, Iso, or Met conditions in subsequent differentiation assay. White arrows indicate mSGOs.
(F) Quantification of mSGO formation efficiency with Iso in self-renewal conditions and further presence of Iso or Met in differentiation conditions. $n \geq 4$ biological replicates (separate patients) per condition and passage mSGO formation efficiency in Iso-Iso conditions set at 100%.
(G) mSGO formation efficiency in Iso-Iso conditions. Each data point is colored relative to the passage from which it was harvested.
(H) Correlation of summed α-amylase and NKCC1 expression at qPCR level with Notch score. Each point represents a different mSGO formation assay.
(I) Whole-mount immunostaining of mSGOs for Notch downstream target HES1 and acinar markers α-amylase and NKCC1.
(J) Whole-mount immunostaining of mSGOs for ADRB1 plus acinar marker α-amylase and NKCC1. Scale bars in (I) and (J) represent 50 μm.

this study. Literature would suggest, however, that murine and human SGO cultures contain a sizable proportion of basal SD cells (Pringle et al., 2019). Our data suggest that a degree of plasticity between basal SD and luminal ID cells can be observed within SGO cultures. Plasticity of the SG has been suggested by ourselves previously in the clinical context of immune checkpoint inhibitor use, where an abundance of ID-like structures was observed, in place of classical saliva-producing acinar cells (Pringle et al., 2020a, 2020b). Plasticity within the acinar cell-ID axes has also been demonstrated (Shubin et al., 2020; Weng et al., 2019). Using lineage tracing in the mouse, the authors suggest that, under homeostatic conditions, acinar cells are replenished mainly by self-renewal of acinar progenitor cells, and subsidized by ductal progenitor cells (Shubin et al., 2020; Weng et al., 2019). However, under conditions of stress (e.g., radiation or injury inducing severe acinar cell loss), acinar cells can be replaced by both acinar and Keratin $7^+$ and Keratin $19^+$ luminal ductal cells, although with a morphology more reminiscent of SDs than IDs (Shubin et al., 2020; Weng et al., 2019). This suggests an acinar cell-ID axis plasticity (Shubin et al., 2020; Weng et al., 2019). A recent paper employing extensive single-cell sequencing of murine SGs through embryonic and adult time points highlighted a proportion of Keratin $14^+$ basal SD cells that were predicted as precursors to ID cells (Hauser et al., 2020). Although by no means conclusively shown in the current study, we suggest that exposure to the Notch ligand JAG1 in human parotid SGO culture induces not only proliferation of luminal ID cells but a possible lineage switch of basal SD into luminal ID cells.

Our data raise two interesting questions. First, we have observed that luminal ID cells remain present in SG tissue of patients taking β-blockers, together with the fact that there was no decrease in SGO formation following addition of the β-blocker Met to healthy SGO cultures. We also saw reduced ADRB1 expression in luminal ID cells in a patient taking β-blockers, suggest perhaps its downregulation under long-term β-blocker use. We hypothesize from these observations that luminal ID cells remain present under β-blocker exposure but are not functional. Second, this unchanged SGO formation efficiency of healthy SGOs with β-blocker addition is in stark contrast to the low yield of primary SGOs obtained from biopsies of patients taking β-blockers. If only luminal ID cells are affected, one could expect the remaining SGO cells to culture as normal. Long-term β-blocker use may affect other SG ductal cell populations, in a manner that remains to be determined. Third, after the wash-out period of β-blockers, the organoid formation efficiency (OFE) was higher than the controls. One can speculate that the Notch pathway, once released from β-blocker-induced blockade, responds with heightened activation status, and therefore OFE. Relatedly, the reversible nature (or not) of β-blocker-induced sicca remains wholly uninvestigated, due to the severe cardiac disturbances often experienced when patients cease taking β-blockers (the β-blocker rebound effect).

The parotid SGs of patients taking β-blockers also still contain acinar cells, even in the face of reduced luminal ID progenitor cell activity. Perhaps, in an effort to compensate for both suppression of acinar cell activity induced via β-blocker use and β-blocker-induced luminal ID progenitor cell inactivity, acinar compartment-based progenitors proliferate to maintain acinar cell number (Aure et al., 2015; Ingalls et al., 2019). Following a similar train of thought, we also acknowledge that only 1% of patients taking β-blockers will experience persistent hyposalivation under β-blocker administration. A multiple-hit model may account for this 1%, whereby not only are the luminal ID cells compromised but additional SGPC populations are too; for example, through chronic inflammation, the effects of hormones, an unknown genetic predisposition, or a natural decline in SG production ability with age (Wiener et al., 2010). In individuals where no additional hit is present, counterpart SGPCs, for example those in the basal SDs, may compensate for the lack of activity of the luminal ID cells, and saliva production continues.

Continuing with the theme of inflammation, β-adrenergic signaling control of luminal ID progenitor cells may prove clinically useful, for example in pSS, for the treatment of hyposalivation. We have recently demonstrated increased potentially senescent ($p16^+$) cells in the basal SD progenitor cell niche in the parotid SG in pSS, and their correlation with pSS patient clinical parameters (Pringle et al., 2019; Wang, et al., 2020b). Stimulation of the luminal ID progenitor cell niche via the Notch pathway, which demonstrates potentially only mild abnormalities in relation to control parotid SGs, and generation of new acinar cells, may provide pSS patients with relief from their xerostomia complaints (Pringle et al., 2020b). Recent reports have demonstrated that adenoviral vectors trophic for SG acinar cells may facilitate delivery of Notch ligands, for example, to the desired cellular niche (Di Pasquale et al., 2020).

We acknowledge that this study may be limited by unavoidable simultaneous patient use of multiple drugs potentially affecting the SG, which may cloud the true nature of the β-adrenergic receptor signal blockade (Tables S1–S3). Additionally, by coincidence, all biopsies analyzed from patients taking medication affecting the β-adrenergic signaling system were female, which may possibly have skewed our results toward a sex-based bias. Through application of β-adrenergic signaling agonists to SG organoids from healthy SGs from male and female donors, we hope to have negated this pitfall.

In summary, we show that the Notch signaling pathway is intricately involved in control of luminal ID cell dynamics in the human parotid SG and promotes the differentiation of SGOs into mSGOs containing acinar cells.

## EXPERIMENTAL PROCEDURES

### Source of SG tissue

For control biopsies, parotid SG tissue was obtained from donors after written informed consent who were treated for a squamous cell carcinoma of the oral cavity, in which an elective head and neck dissection procedure was performed. During this procedure, parotid SG is exposed and partly removed as part of the dissection procedure. The donors did not register complaints of sicca symptoms, and their parotid gland function was assessed by salivary secretion after 2% citric acid stimulation. Three out of 12 patients from whom control biopsies were taken took medications associated with >10% chance of developing sicca complaints, although salivary secretion after 2% citric acid stimulation did not reflect SG dysfunction (Table S1). The parotid tissue was normal by histology and did not contain malignant cells, in line with lack of metastases of oral squamous cell carcinoma into the parotid SG. The parotid tissue of these individuals was considered healthy. The number of biopsies in each experiment is indicated in the legend of graphs.

Parotid biopsies from patients taking β-blockers were taken during routine diagnosis work-up trajectory from patients experiencing sicca complaints of eyes and mouth. After completing the trajectory, these patients were established not to fulfill the American College of Radiology European League Against Rheumatism classification criteria for pSS. The number of biopsies in the corresponding experiment is indicated in the legend of graphs. All patients gave institutional review board consent and approval (medical ethical testing committe [METc] 2016/010). These patients experienced dry mouth complaints (need to drink water to swallow food, frequently awake in night due to dry mouth, avoidance of dry food). Clinical details of these patients can be found in Tables S1 and S2. In essence, these patients did not reveal anti-Sjögren's syndrome antigenA/B (SSA/B) autoantibodies in serum and did not show signs of focal sialadenitis in the SGs.

### Human parotid SGO cultures

The method for SGPC isolation from parotid SG biopsies, and generation and maintenance of SGOs, and the medium used, was as described in our previous publication (Pringle et al., 2019). For drug treatment of SGOs in self-renewal assays, single SGPCs were seeded into Matrigel as previously described (Pringle et al., 2019), and 1 μM Iso (Sigma), 1 μM Met (Sigma), 100 ng/mL JAG1 (Sigma), or 1 μM DAPT (Sigma) were added into SGPC medium where appropriate (Dang et al., 2009). Organoids appeared 2–3 days post seeding of single cells in Matrigel. Medium was supplemented with 0.5 mL of extra medium every 2–3 days of culture. No extra stimulation was administered to SGO cultures from patients taking β-blocker drugs. Ten days after seeding, Matrigel was dissolved by incubation with Dispase enzyme as above. Organoids over 50 μm in diameter were enumerated, cells were processed to a single-cell suspension using 0.05% trypsin-EDTA, and cell number determined. These data were used to generate the organoid formation efficiency, using the following formula:

$$OFE\ [\%] = \frac{Number\ of\ organoids\ harvested\ at\ the\ end\ of\ the\ passage}{Number\ of\ single\ cells\ seeded\ at\ the\ beginning\ of\ the\ passage}\ x\ 100$$

Encapsulation in Matrigel was repeated to generate the next passage. This cycle was repeated four times (four passages). At the end of each passage, an image was captured of the cells, using the Olympus CKX53 microscope and DP2-SAL software. The volume of Iso and Met (both Sigma) were added to form no more than 1% of total media volume, while still maintaining the desired final concentration. Medium was refreshed every 3–4 days (0.5 mL of relevant medium added).

### Human parotid mSGO differentiation

mSGOs are structures derived from SGOs, containing differentiated cells of the SG. In order to make mSGOs, SGOs were first generated in self-renewal conditions, either in control SGPC medium or with additional 1 μM Iso. At the end of the passage, gels were dissociated using Dispase, collected by centrifugation, and re-suspended in 25 μL of SG medium. This suspension was then mixed with 50 μL of growth-factor-reduced Matrigel and deposited as a drop in the center of a 12-well plate. After incubation for 20 min at 37° to solidify, 1 mL of enhanced SG medium was added, with 1 μM Iso, or 1 μM Met, as required. Enhanced SG medium was composed of 20 ng/mL epidermal growth factor (EGF) (Sigma-Aldrich), 20 ng/mL fibroblast growth factor 2 (FGF2) (Sigma-Aldrich), N2 (Invitrogen), 10 mg/mL insulin (Sigma-Aldrich), 1 mM dexamethasone (Sigma-Aldrich), and 10 μM Rho Kinase Inhibitor (Abcam). Cultures were maintained for 14 days, and medium was supplemented at day 3 and day 6 (0.5 mL of medium volume added). mSGO formation efficiency was calculated by quantifying the number of structures with at least three branches terminating in round end buds. mSGOs were enumerated as a proportion of seeded number of SGOs.

### Further methods

Quantification and statistical analysis, qPCR, RNA-seq analysis, paraffin section staining, whole-mount staining, frozen section staining, cell smear staining protocols, and antibody sources can be found in the supplementary file.

## DATA AVAILABILITY STATEMENT

The data that support the findings of this study are available from the corresponding author upon reasonable request. The accession number for the RNA-seq data reported in this paper is NCBI Sequence Reads Archive: accession number PRJNA506620.

## SUPPLEMENTAL INFORMATION

Supplemental information can be found online at https://doi.org/10.1016/j.stemcr.2021.09.015.

## AUTHOR CONTRIBUTIONS

X.W., collection and/or assembly of data, data analysis and interpretation, manuscript writing and analysis, and final approval of manuscript; P.S.M., data analysis and interpretation, and final approval of manuscript. J.H.T, collection and/or assembly of data, data analysis and interpretation, and final approval of manuscript. A.S., data analysis and interpretation, and final approval of manuscript. G.P., data analysis and interpretation, and final approval of manuscript. F.K.L.S., provision of study material of patients, and final approval of manuscript. A.V., conception and design, and final approval of manuscript. H.B., final approval of manuscript. R.P.C., conception and design, manuscript writing, and final approval of manuscript. F.G.M.K., conception and design, manuscript writing, and final approval of manuscript. S.P., conception and design, data analysis and interpretation, manuscript writing, and final approval of manuscript.

## CONFLICTS OF INTEREST

The authors declare no competing interests.

## ACKNOWLEDGMENTS

We gratefully acknowledge the maxillofacial surgical teams at the University Medical Center Groningen (Prof. Max Witjes) and Medical Center Leeuwarden (Dr. Eric van der Meij, Dr. Christiaan Krabbe and Dr. Visscher) for their continuous collaboration and coordination of tissue collection. Financial support: this research was funded by a China Scholarship Council grant (201606220074), Dutch Arthritis Foundation Translational Research Grant (T015-052), and a Dutch Arthritis Foundation Long Term Project Grant (LLP-29).

Received: April 12, 2021
Revised: September 20, 2021
Accepted: September 21, 2021
Published: October 21, 2021

## REFERENCES

Aure, M.H., Konieczny, S.F., and Ovitt, C.E. (2015). Salivary gland homeostasis is maintained through acinar cell self-duplication. Dev. Cell *33*, 231–237.

Bloch, M. (2016). Worldwide prevalence of hypertension exceeds 1.3 billion- ClinicalKey. Am. Soc. Hypertens. *10*, 753–754.

Clevers, H. (2016). Modeling development and disease with organoids. Cell *165*, 1586–1597.

Cowman, R.A., et al. (1994). Effects of beta-adrenergic antagonists on salivary secretory function in individuals of different ages. J. Gerontol. *49*, B208–B214. https://doi.org/10.1093/geronj/49.5.B208.

Dang, H., et al. (2009). Role for notch signaling in salivary acinar cell growth and differentiation. Dev. Dyn. *238*, 724–731.

Djukić, L., et al. (2015). The effects of anti-hypertensives and type 2 diabetes on salivary flow and total antioxidant capacity. Oral Dis. *21*, 619–625. https://doi.org/10.1111/odi.12325.

Glick, A., Sista, V., and Cleverick, J. (2020). Oral manifestations of commonly prescribed drugs - American Family Physician. Am. Fam. Physician *102*, 613–621.

Hand, A.R., and Ho, B. (1985). Mitosis and hypertrophy of intercalated duct cells and endothelial cells in the isoproterenol-treated rat parotid gland. J. Dent Res. *64*, 1031–1038.

Hauser, B.R., et al. (2020). Generation of a single-cell RNAseq atlas of murine salivary gland development. iScience *23*, 101838. https://doi.org/10.1016/j.isci.2020.101838.

Humphreys-Beher, M., et al. (1987). Isoproterenol-mediated parotid gland hypertrophy is inhibited by effectors of 4 beta-galactosyltransferase. J. Biol. Chem. *262*, 11706–11713.

Hwang, S.-M., et al. (2014). Role of LPA and the Hippo pathway on apoptosis in salivary gland epithelial cells. Exp. Mol. Med. *46* (12), e125.

Ingalls, M.H., et al. (2019). Intrinsic mitotic activity supports the human salivary gland acinar cell population. FEBS Lett. *94*, 376–382.

Johnson, D.A., and Cortez, J.E. (1988). Chronic treatment with beta adrenergic agonists and antagonists alters the composition of proteins in rat parotid saliva. J. Dental Res. *67*, 1103–1108. https://doi.org/10.1177/00220345880670080801.

Kane, S.P. (2019). Clinical tools and calculators for medical professionals - ClinCalc. https://clincalc.com/DrugStats/.

Knox, S.M., et al. (2010). Parasympathetic innervation maintains epithelial progenitor cells during salivary organogenesis. Science *329*, 1645–1647.

Knox, S.M., et al. (2013). Parasympathetic stimulation improves epithelial organ regeneration. Nat. Commun. *4*, 1494. https://doi.org/10.1038/ncomms2493.

Kwak, M., et al. (2018). c-Kit+ cells in adult salivary glands do not function as tissue stem cells. Sci. Rep. *8*, 14193.

van Luijk, P., et al. (2015). Sparing the region of the salivary gland containing stem cells preserves saliva production after radiotherapy for head and neck cancer. Sci. Transl. Med. *7*, 305ra147. https://doi.org/10.1126/scitranslmed.aac4441.

Maimets, M., et al. (2016). Long-term in vitro expansion of salivary gland stem cells driven by Wnt signals. Stem Cell Rep. *6*, 150–162.

de Matos, L.F., et al. (2010). Relationships of beta-blockers and anxiolytics intake and salivary secretion, masticatory performance and taste perception. Arch. Oral Biol. *55*, 164–169. https://doi.org/10.1016/j.archoralbio.2009.11.011.

Matsuo, R., et al. (2000). Reflex secretion of proteins into submandibular saliva in conscious rats, before and after preganglionic sympathectomy. J. Physiol. *527*, 175–184. https://doi.org/10.1111/j.1469-7793.2000.00175.x.

Nederfors, T. (1996). Xerostomia: prevalence and pharmacotherapy. With special reference to beta-adrenoceptor antagonists. Swed Dent J. Suppl. *116*, 1–70.

Di Pasquale, G., et al. (2020). Transduction of salivary gland acinar cells with a novel AAV vector 44.9. Mol. Ther. - Methods Clin. Dev. *19*, 459–466. https://doi.org/10.1016/j.omtm.2020.10.006.

Peng, X., et al. (2017). Role of glial-cell-derived neurotrophic factor in salivary gland stem cell response to irradiation. Radiother.

Oncol. *124*, 448–454. https://doi.org/10.1016/J.RADONC.2017.07.008.

Pringle, S., et al. (2019). Salivary gland stem cells age prematurely in primary Sjögren's syndrome. Arthritis Rheumatol. *71*, 133–142.

Pringle, S., et al. (2020a). Checkpoint inhibition-induced sicca: a type II interferonopathy? Clin. Exp. Rheumatol. *38*, 253–260.

Pringle, Sarah, et al. (2020b). Lack of conventional acinar cells in parotid salivary gland of patient taking an anti-PD-L1 immune checkpoint inhibitor. Front. Oncol. *10*, 420.

Proctor, G.B., and Carpenter, G.H. (2007). Regulation of salivary gland function by autonomic nerves. Auton. Neurosci.-Basic Clin. *133*, 3–18. https://doi.org/10.1016/j.autneu.2006.10.006.

Proctor, G.B., Garrett, J.R., and Asking, B. (1988). Influences of short-term sympathectomy on the composotion of proteins in rat parotid saliva. Q. J. Exp. Physiol. *73*, 139–142. https://doi.org/10.1113/expphysiol.1988.sp003113.

Purushotham, K.R., et al. (1992). A novel mechanism for isoprenaline-stimulated proliferation of rat parotid acinar cells involving the epidermal growth factor receptor and cell surface galactosyltransferase. Biochem. J. *284*, 767–776. https://doi.org/10.1042/bj2840767.

Selye, H., Veilleux, R., and Cantin, M. (1961). Excessive stimulation of salivary gland growth by isoproterenol. Science *133*, 44–45.

Shubin, A.D., et al. (2020). Stress or injury induces cellular plasticity in salivary gland acinar cells. Cell Tissue Res. *380*, 487–497.

Speirs, R.L., et al. (1974). The influence of sympathetic activity and isoprenaline on the secretion of amylase from the human parotid gland. Arch. Oral Biol. *19*, 747–752.

Srinivasan, P.P., et al. (2016). Primary salivary human stem/progenitor cells undergo microenvironment-driven acinar-like differentiation in hyaluronate hydrogel culture. Stem Cells Transl. Med. *6*, 110–120.

van Stegeren, A., et al. (2006). Salivary alpha amylase as marker for adrenergic activity during stress: effect of betablockade. Psychoneuroendocrinology *31*, 137–141.

Wang, X., Bootsma, H., de Koning, J., et al. (2020a). Novel approaches for rescuing function of the salivary gland epithelium in primary Sjögren's syndrome. Clin. Exp. Rheumatol. *38*, 261–270.

Wang, X., Bootsma, H., Terpstra, J., et al. (2020b). Progenitor cell niche senescence reflects pathology of the parotid salivary gland in primary Sjögren's syndrome. Rheumatology *59*, 3003–3013. https://doi.org/10.1093/rheumatology/keaa012.

Weng, P.-L.L., et al. (2018). Limited regeneration of adult salivary glands after severe injury involves cellular plasticity. Cell Rep. *24*, 1464–1470.e3. https://doi.org/10.1016/j.celrep.2018.07.016.

Weng, P., Luitje, M.E., and Ovitt, C.E. (2019). Cellular plasticity in salivary gland regeneration. Oral Dis. *25*, 1837–1839. https://doi.org/10.1111/odi.13205.

Wiener, R., et al. (2010). Hyposalivation and xerostomia in dentate older adults. J. AM. Dent Assoc. *141*, 279–284.

Wolff, A., et al. (2017). A guide to medications inducing salivary gland dysfunction, xerostomia, and subjective Sialorrhea: a systematic review sponsored by the world workshop on oral medicine VI. Drugs R&D *17*, 1–28.

Xiao, N., et al. (2014). Neurotrophic factor GDNF promotes survival of salivary stem cells. J. Clin. Invest. *124*, 3364–3377.

Yeh, C.K.K., et al. (2012). Cellular signals underlying β-adrenergic receptor mediated salivary gland enlargement. Differentiation *83*, 68–76.

Zhou, Y., et al. (2015). Early gene expression in salivary gland after isoproterenol treatment. J. Cell Biochem. *116*, 431–437.

**Stem Cell Reports, Volume *16***

## Supplemental Information

# β-Adrenergic signaling induces Notch-mediated salivary gland progen-itor cell control

**X. Wang, P. Serrano Martinez, J.H. Terpstra, A. Shaalan, G.B. Proctor, F.K.L. Spijkervet, A. Vissink, H. Bootsma, F.G.M. Kroese, R.P. Coppes, and S. Pringle**

# Notch-mediated salivary gland progenitor cell inhibition by β-blockers

# Supplementary Figures

## Fig. S1

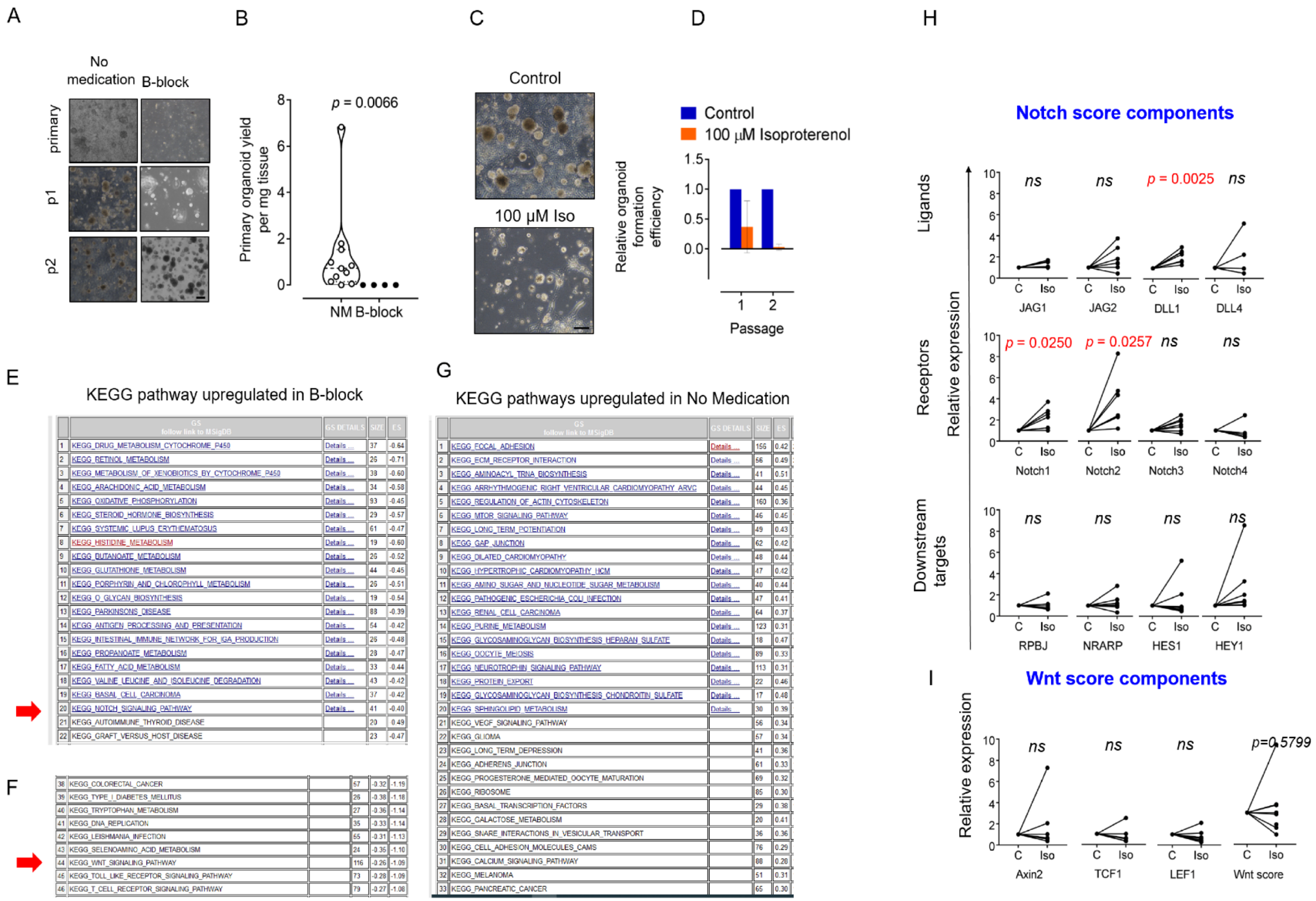

**Fig. S2.**

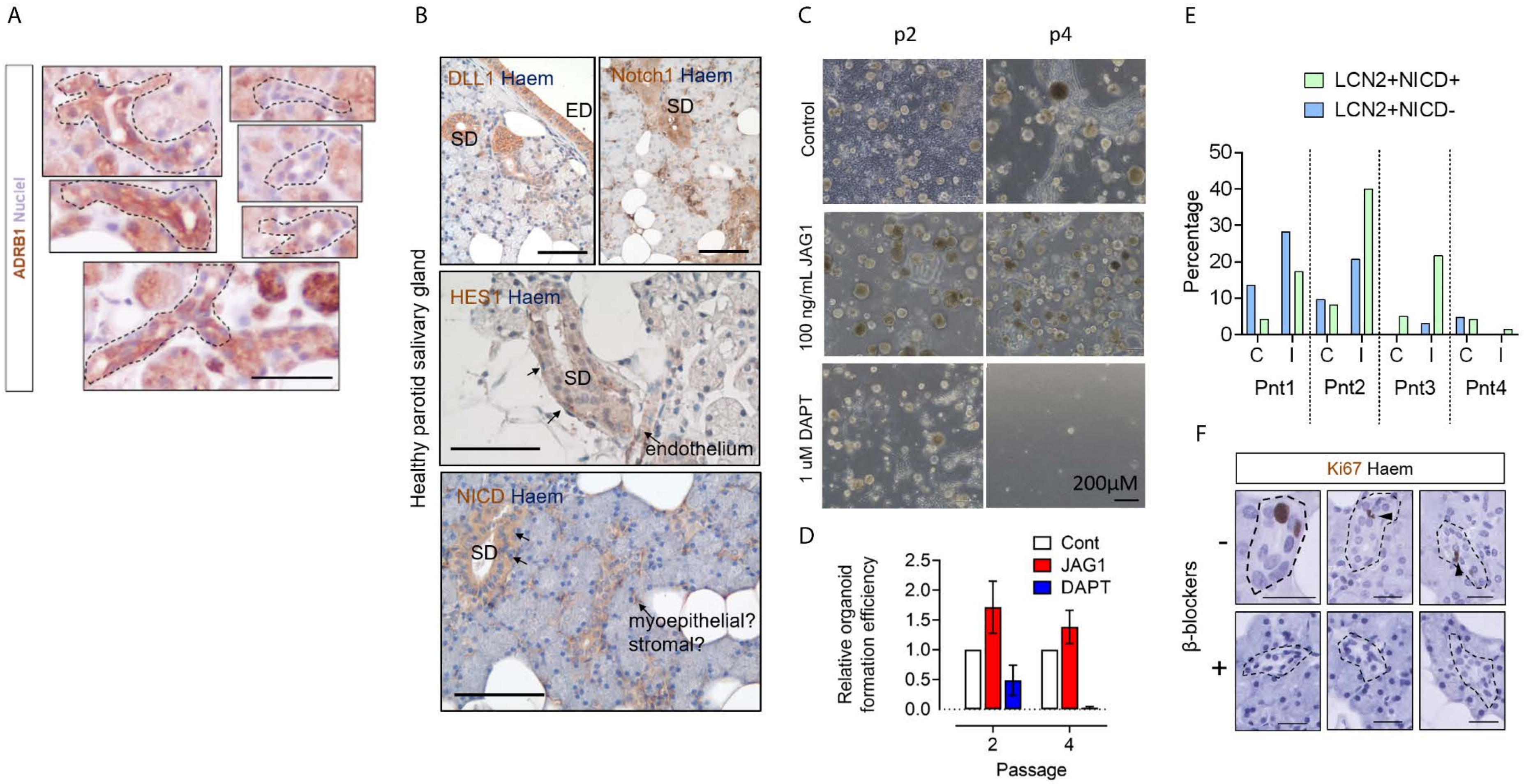

A
ADRB1 Nuclei
B
DLL1 Haem
ED
SD
Notch1 Haem
SD
HES1 Haem
SD
endothelium
NICD Haem
SD
myoepithelial?
stromal?
Healthy parotid salivary gland
C
p2
p4
Control
100 ng/mL JAG1
1 uM DAPT
200µM
D
Cont
JAG1
DAPT
Relative organoid formation efficiency
2.5
2.0
1.5
1.0
0.5
0.0
2
4
Passage
E
LCN2+NICD+
LCN2+NICD-
Percentage
50
40
30
20
10
0
C
I
Pnt1
Pnt2
Pnt3
Pnt4
F
Ki67 Haem
β-blockers
-
+

**Fig. S3**

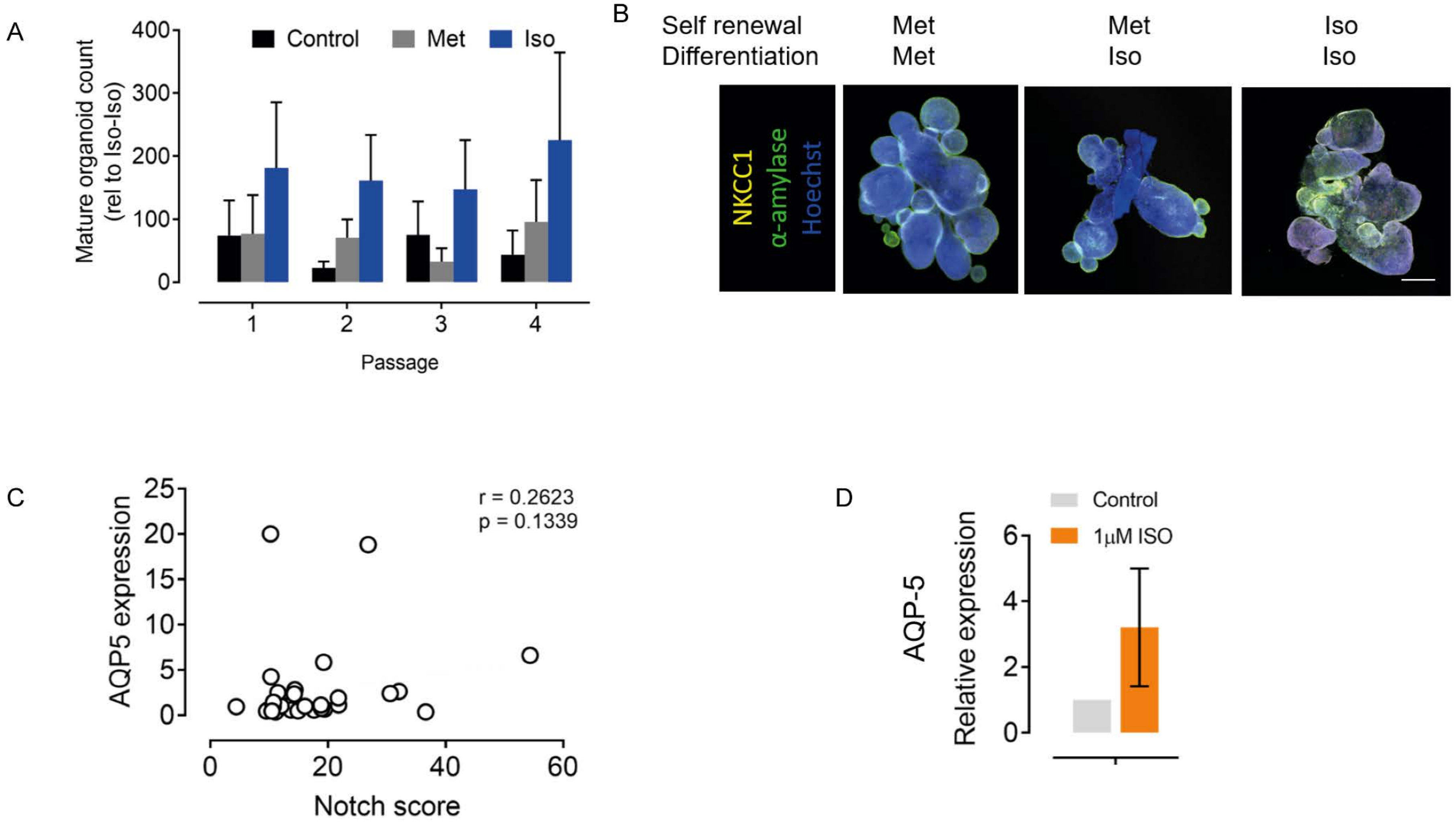
A
Mature organoid count
(rel to Iso-Iso)
Control
Met
Iso
400
300
200
100
0
1
2
3
4
Passage
B
Self renewal
Differentiation
Met
Met
Met
Iso
Iso
Iso
NKCC1
α-amylase
Hoechst
C
AQP5 expression
r = 0.2623
p = 0.1339
25
20
15
10
5
0
0
20
40
60
Notch score
D
AQP-5
Relative expression
Control
1μM ISO
6
4
2
0

# Supplementary Figure legends

**Fig. S1.** *Salivary gland organoid yield from biopsies of a patient taking β-blocking drugs was initially low. RNASeq analysis shows upregulation of Notch signaling when cultures following β -blocker use recover in organoid formation efficiency*. *The Notch pathway in salivary gland organoids is activated by isoproterenol, whereas the Wnt pathway is not.* A) Phase contrast microscopy of organoid cultures generated from biopsies harvested from patients taking β-blocking drugs (B-block) in primary cultures, passage 1 and passage 2, compared to biopsies from patient not taking any medication (No medication). B) Quantification of organoid forming efficiency in primary culture. Each data point represents a separate patient. NM = No medication. C) Phase contrast microscopy of SGPC cultures in control and 100 µM isoproterenol conditions. D) Quantification of 100 µM Isoproterenol supplementation. $n$ = 3 separate patient isolations. Bar represents mean, error bars represent standard deviation. E) Gene Set Enrichment Analysis (GSEA) using KEGG pathway gene sets, and identifying pathways upregulated in p2 organoids from patient taking a *β*-blocking drug. Red arrow denotes ranking of Notch signaling pathway (left panel). F) Position of Wnt pathway in upregulated genes p2 organoids from patient taking a *β*-blocking drug. G) GSEA identifying pathways upregulated in healthy control organoids. Tables in E show number of genes in gene set (size), expression score (ES), normalized ES (NES). $p$ values are not shown, due to statistical invalidity of comparing one biopsy from a patient taking β-blocking drugs to 6 controls. H) Relative expression of Notch score components, grouped by ligands, receptors and downstream targets. I) Relative expression of Wnt score components and total Wnt score. In all figures, paired $t$-testing was performed. Each paired data set represents a separate patient isolation. Raw $p$-values are given when significant. ns = no significance.

**Fig. S2.** *ADRB1 expression decreases in some intercalated ducts following β-blocker use*. *DLL1, Notch1, HES1 and NICD are expressed by parotid SG striated duct cells. Incubation of human parotid SGOs with the Notch ligand JAG1 induces a subtle increase in the number of SGOs, whereas inhibiting the Notch pathway reduces all organoid formation* A) Expression of ADRB1 in intercalated ducts (outlined with dashed lines) in a patient following β-blocking drug use. All images are from the same tissue sample. Scale bar = 50 µM. B) Healthy parotid salivary gland immunostained for DLL1,

Notch1 and HES1, showing expression of DLL1 and Notch1 in both basal and luminal striated duct layers. DLL1 was additionally expressed in excretory ducts, Notch1 in scattered stromal cells, and HES1 in basal striated duct cells (arrows) and endothelial cells. C) Phase contrast microscopy of SGOS cultures at passages 2 and 4, in control, 100 ng/mL JAG1 and 1 μM DAPT conditions. D) Quantification of control, JAG1 and DAPT incubations. $n$ = ⩾5 separate patient isolations at each passage and for each group. E) Percentage of SGO cells expressing LCN2 and NICD when cultured in control conditions, or with isoproterenol.  Pnt = Patient. F) Ki67 staining of tissue from a patient taking β-blockers (+) and in control SG tissue (-).

**Fig. S3** *Supplementary human parotid mature SGO formation data* A) Quantification of mSGO formation efficiency without isoproterenol exposure in self-renewal assay conditions. $n$ = 4, 9, 5 and 4 for passages 1,2,3 and 4, respectively. Each data point is a separate biological replicate B) Whole mount staining of mSGOs for expression of NKCC1 and α-amylase in culture conditions containing metoprolol or isoproterenol, as indicated. C) Correlation between AQP5 expression and Notch scores in mSGO differentiation assays. Data points represent the expression of AQP5 from 2 patients from passages 1 to 4 in differentiation. D) Expression of AQP5 in self-renewal assays exposed to 1μM isoproterenol. $n$=7, each data point is a separate biological replicate.

# Supplementary Tables

## Supplementary Table 1

List of medications taken by non ß-blocker-exposed ‘healthy SG’ donors, and additional medication taken by patients taking β-blockers.

| Healthy salivary gland donor | Medications used |
|---|---|
| 1 | Atorvastatin, carbasalate calcium, clopidogrel, ezetimib, pantoprazol, paracetamol, perindopril** |
| 2 | Aspirin, Furosemide*, pantoprazol, amlodipine, candesartan, diazepam*, diclofenac |
| 3 | Spiriva, vitamine B12 injections |
| 4 | Acetylcysteine, omeprazol, brimonidine* |
| 5 | Simvastatin, chloramphenicol, dexamethasone eye drops, diclofenac eye drops, |
| 6 | Hydrochlorothiazide |
| 7 | Diclofenac, levothyroxine, omeprazol |
| 8 | None |
| 9 | None |
| 10 | None |
| 11 | Dextran hypromellose eyecream, triptoreline, paracetamol |
| 12 | Propranolol* |
| Patients taking β-blockers | Additional medications used |
| 1 | Seretide, Salbutamol, Euthyrax, Omeprazole, vit-D, Ascal, Atorvastatin, paracetamol codeine, Fexofenadine, eye cream, Dermovate vaginal, artificial tears. |
| 2 | talbutamine, metformine, hydrochlorothiazide, lisinopril*, prednisolone, acenocoumarol, simvastatine, slow K, Omeprazol, psyllium vezels, brinzolamide, artelac, vidisec carbogel, Budesonide (anti-inflammatory). |
| 3 | Omeprazole |
| 4 | Plaquenil, Bromazepam, Vidisec, Carbogel, Aprovel. |
| 5 | Triamtereen, Tiotropium, Omeprazole, Naproxen, Irbesartan, Insuline glargine, Dextra 70/hypromellose oogdruppels, Ciclesonide aerosol |
| 6 | Ezetimibe |

**Table S1.** Details of medication used by representative patients donating healthy salivary gland biopsies, and those taking β-blockers. . Medications indicated with asterixes have potential for interaction with salivary gland function according to Wolff et al, Drugs R D., 2017 17(1): 1-28.  * High / moderate levels of evidence, ** weak evidence.

**Supplementary Table 2**

Clinical characteristics of patients taking ß-adrenoreceptor blocking medication analyzed in this study.

| Patient | β-blocking medication | Age | Sex | Resting secretion speed ml/min | | | Stimulated secretion speed ml/min | | | Analysis performed | Used in |
|---|---|---|---|---|---|---|---|---|---|---|---|
| | | | | PR | PL | Sub+SubL | PR | PL | Sub+SubL | | |
| 1 | Metoprolol Citalopram* | 62 | F | 0 | 0.004 | 0.08 | 0.08 | 0.08 | 0.21 | SGO culture | FigS1, Fig1 |
| 2 | Metoprolol Amitriptyline* | 74 | F | 0 | 0 | 0.02 | 0.03 | 0.02 | 0.02 | SGO culture | FigS1, Fig1 |
| 3 | Metoprolol Citalopram* | 58 | F | 0 | 0 | 0.04 | 0 | 0 | 0.07 | SGO culture | FigS1, Fig1 |
| 4 | Duloxcetine Escitalopram | 48 | F | 0.01 | 0.01 | 0.24 | 0.01 | 0.26 | 0.39 | SGO culture & RNASeq | FigS1, Fig1 |
| 5 | Metoprolol | 66 | F | 0.1 | 0.09 | 0.44 | 0.18 | 0.17 | 0.42 | Immunohistochemistry | Fig1B-D |
| 6 | Metoprolol | 62 | F | 0 | 0 | 0.01 | 0.01 | 0.02 | 0.05 | Immunohistochemistry | Fig1B-D |

**Table S2.** Clinical characteristics of patients taking ß-blocking medication, from which biopsies were studied. Abbreviations: PR = parotid right; PL – parotid left; Sub+SubL = submandibular and sublingual salivary gland secretion combined; SGO = salivary gland organoid culture. Stimulated secretion speeds were measured using citric acid. Additional drugs capable of causing hyposalivation according to Wolff et al, (Drugs R D., 2017 17(1): 1-28) are marked with an asterix. * High / moderate levels of evidence

**Supplementary Table 3**

| Gene name | Gene Symbol | Forward primer | Reverse primer | Amp size |
|---|---|---|---|---|
| glyceraldehyde-3-phosphate dehydrogenase | GAPDH | tcttttgcgtcgccagccgag | cccgttctcagccttgacggtg | 234 |
| Jagged1 | JAG1 | aggccgttgctgacttagaa | gcagaagtgggagctcaaag | 230 |
| Jagged2 | JAG2 | gtcaaggtggagacggttgt | tggtagagcacgtccttgtg | 250 |
| Delta-like ligand 1 | DLL1 | tgtgcctcaagcactaccag | ttctgttgcgaggtcatcag | 230 |
| Delta-like ligand 4 | DLL4 | acctttgggtgtctgtctgg | acttttggaaacacggatgc | 209 |
| Notch Receptor 1 | NOTCH1 | actgtgaggacctggtggac | ttgtaggtgttggggaggtc | 196 |
| Notch Receptor 2 | NOTCH2 | atgactgccctaaccacagg | ccagccgttgacacatacac | 206 |
| Notch Receptor 3 | NOTCH3 | atcttggggcctaaagaga | gactgagagggtgggtggta | 202 |
| Notch Receptor 4 | NOTCH4 | ctaggggctcttctcgtcct | caacttctgcctttggcttc | 178 |
| NOTCH regulated ankyrin repeat protein | NRARP | gactcaattcgaacccgaaa | acttccatgaaggggaaacc | 220 |
| Recombination signal binding protein for immunoglobulin kappa J region | RBPJ | cgcattattggatgcagatg | caggaagcgccatcatttat | 171 |
| hes family bHLH transcription factor 1 | HES1 | ctctcttccctccggactct | aggcgcaatccaatatgaac | 186 |
| hes related family bHLH transcription factor with YRPW motif 1 | HEY1 | cgaggtggagaaggagagtg | ctgggtaccagccttctcag | 177 |
| Aquaporin-5 | AQP5 | actgggttttctgggtaggg | gtggtcagctccatggtctt | 184 |
| α-amylase | AMY | tgtcagggctgagtgttctg | ttccggtataaatgcgaacc | 198 |
| Na-K-2Cl cotransporter 1 | NKCC1 | tcagtcagccatacccaaagg | cagtggcatctcgaacaacac | 107 |
| axis inhibition protein 2 | Axin2 | cctgccaccaagacctacat | cttcattcaaggtggggaga | 241 |
| transcription factor 1 | TCF1 | gcagggtctagaaggctgtg | acctgctctaccagccagaa | 242 |
| lymphoid enhancer binding factor 1 | LEF1 | aacatggtggaaaacgaagc | gggttggcagtgattgtctt | 201 |

**Supplementary Table 4**

| Target | Target species | Abbrev. | Host | Supplier | Catalog # | Clone | Dilution |
|---|---|---|---|---|---|---|---|
| **Primary antibodies** | | | | | | | |
| β-1 adrenergic receptor | human | ADRB1 | Rabbit | ThermoFisher Scientific | PA1-049 | Polyclonal | 1:50 |
| DLL1 | human | - | Rabbit | Novus Biologicals | NBP2-27088SS | Polyclonal | 1:100 |
| notch 1 | human | - | Mouse | Santa Cruz Biotechnology | 11-651-C025 | mN1A | 1:1000 |
| HES1 | human | - | Rabbit | Abcam | ab71559 | Polyclonal | 1:50 |
| Notch intracellular domain | Human | NICD | Rabbit | Abcam | Ab8387 | Polyclonal | 1:1000 |
| cytokeratin 7 | human | K7 | Mouse | Merck | MAB3226 | RCK105 | 1:100 |
| Ki67 | human | - | Rat | ThermoFisher Scientific | 14-5698-82 | SolA15 | 1:100 |
| NKCC1 | human | - | Goat | Abcam | ab99558 | Polyclonal | 1:75 |
| α-amylase | human | - | Sheep | ThermoFisher Scientific | PA1-85176 | Polyclonal | 1:50 |
| cytokeratin 14 | human | K14 | Mouse | Merck | CBL197 | LL002 | 1:100 |
| Lipocalin-2 | Human | LCN2 | Rabbit | Merck | HPA002695 | Polyclonal | 1:50 |
| smooth muscle actin | human | SMA | Mouse | DAKO | M085129-2 | 1A4 | 1:100 |
| aquaporin 5 | human | AQP5 | Rabbit | Abcam | ab92320 | EPR3747 | 1:200 |
| **Secondary antibodies** | | | | | | | |
| Rabbit and  mouse IgG UltraVision HRP polymer | | | N/A | DAKO | TL-125-HL | N/A | Ready-to-use |
| Mouse and rabbit IgG, AP and HRP conjugated, double staining kit | | | N/A | ThermoFisher Scientific | TL-012-MARH | N/A | Ready-to-use |
| Mouse IgG (H+L) Alexa Fluor 488 conjugated | | | Goat | ThermoFisher Scientific | A-11001 | N/A | 1:1500 |
| Rabbit IgG (H+L) Alexa Fluor Plus 594 conjugated | | | Donkey | ThermoFisher Scientific | A32754 | N/A | 1:1500 |
| Goat IgG (H+L) Alexa Fluor Plus 647 conjugated | | | Donkey | ThermoFisher Scientific | A32849 | N/A | 1:1500 |
| Rat IgG (H+L) Alexa Fluor 647 conjugated | | | Goat | ThermoFisher Scientific | A-21247 | N/A | 1:1500 |
| Sheep IgG–FITC conjugated | | | Donkey | Merck | F7634-1ML | N/A | 1:40 |
| Mouse IgG, biotin conjugated | | | Rabbit | DAKO | E0433 | N/A | 1:300 |
| Rabbit IgG, biotin conjugated | | | Goat | DAKO | E0432 | N/A | 1:300 |

# Supplementary Methods

### Quantification and statistical analysis

Methods used for quantification are described in relevant sections. Data was tested for normality using Shapiro-Wilk test. Two-Way ANOVAs were used to test for significant differences, if n numbers were matched across groups and data was parametric. Dunnett's post-hoc testing was used for multiple comparisons, to establish between which groups significant differences lay. If uneven *n* numbers were present across groups, a mixed effects model was used, with subsequent Tukey's post-hoc testing for multiple comparisons. If data was non-parametric, a Kruskal-Wallis test with Dunn's post-hoc testing was used. Adjusted *p*-values for each analysis are given in the relevant text section. Statistical analysis was performed using GraphPad Prism 8.02.

### qPCR analysis

Total RNA as was extracted from cultured cells as appropriate using the RNeasy Microkit (Qiagen), including DNase incubation, as per manufacturer's instructions. One µg of total RNA was reverse transcribed to cDNA using 0.5 µg oligo(dT)$_{15\text{-}18}$ primers, 1.0 mM dNTPs, 1X Reaction Buffer, 20U Ribolock and 200 U of RevertAid Reverse Transcriptase (all Thermo Fischer Scientific), in a total volume of 20 µL per reaction. cDNA product was diluted ten-fold in water and used at this concentration for qPCR. qPCR was performed using SsoAdvanced Universal SYBR Green qPCR Mastermix (Biorad), with primers at a final concentration of 500 nM from a 10 µM stock. 2.5 µL of diluted cDNA was used per reaction, and all reactions were performed in triplicate, in a total volume of 10 µL. Primer sequences can be found in Supplementary Table 3. A 2-step qPCR cycle with the BioRad iCycler qPCR machine was used for target amplification according to SSoAdvanced Universal SYBR Green Mastermix instructions, and CFX Manager software for analysis. Expression of all 12 members of the Notch pathway, relative to GAPDH, was summed to derive the 'Notch score'. The Wnt score was derived from addition of relative expression of 3 Wnt pathway genes.

### RNASeq analysis of SGOs

Total RNA was extracted from salivary gland organoids from a patient taking β-blockers and 6 controls by using Absolutely RNA Miniprep kit (Agilent Technologies, CA. Cat: 400800) followed the manufacturer's recommendations. The integrity of RNA was

examined by Agilent 2100 bioanalyzer. Subsequent sequencing was performed by using SMART-Seq v4 Ultra Low Input RNA Kit (Clontech, Cat: 634890) and Nextera XT DNA Library Prep Kit (Illumine, Cat: FC-131-1096) followed manufacturer’s recommendations. Prepared DNA libraries were sequenced on HiSeq 2500 System. Gene set enrichment analysis (GSEA) was performed using GSEA software version 4.0.2, using a defined KEGG gene set for pathway analysis (tp.broadinstitute.org://pub/gsea/gene_sets/c2.cp.kegg.v6.2.symbols.gmt). Owing to *n*=1 nature of biopsy analyzed, no statically valid calculation of p values could be performed. Data can be accessed via the NCBI Sequence Reads Archive (accession number PRJNA506620).

**Paraffin section staining**

For immunostaining of human tissue, formalin-fixed paraffin embedded 4µm sections of human parotid salivary gland underwent antigen retrieval with EDTA buffer (pH = 8) for 15 min. A double staining kit from Thermofisher was used, according to manufacturer’s instructions. Primary antibodies and concentrations are detailed in the Key Resources table. Sections were imaged using the Olympus BX50 microscope. After dehydration, the tissue was paraffin-embedded and sectioned at 4um thickness. The tissue sections were dewaxed, boiled for 8 min in pre-heated 10mM citric acid (Sigma-Aldrich) retrieval buffer pH 6.0, containing 0.05% Tween20. Primary and secondary antibodies were as in Supplementary Tables 4 Labelling was amplified using an avidin-biotin-horse radish peroxidase complex (VECTASTAIN® Elite® ABC HRP Kit Peroxidase, Standard, PK-6100) and the diaminobenzidine (DAB) chromogen (SIGMAFAST™ 3,3 DAB Tablets). Nuclear staining was performed with hematoxylin. Images were acquired with Leica DM6 B microscope using LAS X software.

**Whole mount staining mSGOs**

Samples were released from culture gels using 1mg/mL Dispase as per passaging protocol and fixed in cold 4 % PFA for 40 minutes. Samples were then washed in PBS and blocked in 1 % BSA 0.5 % Triton for 15 min at room temperature (RT). Samples were allowed to settle, and blocking buffer removed carefully. Primary antibodies were then added at concentrations stated in Supplementary Table 4, in 1 % BSA 0.5 % Triton, and incubated overnight at 4 ºC . To wash SGOs/mSGOs, staining buffer was removed as much as possible and replaced with fresh staining buffer, and left to

incubate for 1 hour at RT. This was repeated 3 times. Secondary antibodies were added at final concentrations of 1:1500 in 0.2 % BSA, 0.5 % Triton, and again incubated overnight at 4 ºC (supplementary Table 6). Washing steps were repeated as above. After the final wash, as much washing buffer was removed as possible, and structures incubated for 20 minutes at RT in clearing solution, before removal from staining well and mounting according to Dekker et al protocol (40). Structures were imaged on the Leica TCS SP8 microscope, including composition of maximum projection and tiled images.

**Frozen sections SGOs and staining**

SGOs harvested from self-renewal plates in the control and isoproterenol treated groups were fixed in cold 4 % PFA for 40 minutes, following by washing with 1%BSA/PBS twice. After that, SGOs were incubated in 20% sucrose for 2 hours, until SGOs sunk to the bottom of the eppendorf. The majority of the sucrose was removed, leaving approximately 50µL left. The SGOs were then placed in a mould filled with approximately 150µL Tissue-Tek® O.C.T.™ Compound and incubated at -20 ºC for at least 20 min. 5 µm thickness of sections SGOs were prepared. SGOs sections were firstly fixed with 4% PFA for 5 min, then incubated with primary antibodies (ADRB1, cytokeratin 7, HES1 and Ki67, as shown in the Supplementary Table 4). Slides were carefully washed and secondary antibodies added as shown in the Supplementary Table 6, and counterstained with Hoechst for visualizing the nuclei. Structures were imaged on the Leica TCS SP8 microscope, including composition of maximum projection and tiled images.

**Cell smear staining protocol**

Samples to be analyzed were processed to single cells using Dispase and Trypsin as described in the self-renewal protocol. Cells were fixed in 4 % PFA for 20 minutes at RT, before washing with PBS, and resuspending in 15uL PBS. Gelatin-coated microscopy slides were prepared by submerging slides in a 0.3 % (w/v) solution of type A gelatin (275 Bloom; Sigma) containing 0.05% (w/v) chromium potassium sulphate (Sigma), at 40-50 ºC degrees Celsius. Slides were allowed to dry, and cell suspension smeared over the surface of the slide using a P100 pipette tip. When dried, smeared cells were circled with a wax pen, and cells incubated with primary anti LCN2, or NICD antibodies (Supplementary Table 4) overnight at 4 ºC. Following washes in PBS, cells

were incubated in appropriate secondary antibodies and subsequent flourophores provided in the Tyramide Signal Amplification Superboost kit (Thermofisher) washed again in PBS, and mounted in Faramount mounting medium. Cell smears were imaged using the Leica TCs SP8 confocal microscope. At least 4 fields of randomly selected microphotographs under 630x magnification in each group were quantified. The proportion of $LCN2^{+}$, $NICD^{+}$ and $LCN2^{+}NICD^{+}$ cells were calculated by the amount of $LCN2^{+}$, $NICD^{+}$ and $LCN2^{+}NICD^{+}$ cells divided by total cells (determined by the number of nuclei).
